# Molecular classification of urothelial carcinoma: global mRNA classification versus tumour‐cell phenotype classification

**DOI:** 10.1002/path.4886

**Published:** 2017-03-28

**Authors:** Gottfrid Sjödahl, Pontus Eriksson, Fredrik Liedberg, Mattias Höglund

**Affiliations:** ^1^ Division of Urological Research, Department of Translational MedicineLund University, Skåne University HospitalMalmöSweden; ^2^ Division of Oncology and Pathology, Department of Clinical SciencesLund UniversityLundSweden

**Keywords:** bladder cancer, molecular classification, tumour‐cell phenotypes, pseudo‐differentiation

## Abstract

Global mRNA expression analysis is efficient for phenotypic profiling of tumours, and has been used to define molecular subtypes for almost every major tumour type. A key limitation is that most tumours are communities of both tumour and non‐tumour cells. This problem is particularly pertinent for analysis of advanced invasive tumours, which are known to induce major changes and responses in both the tumour and the surrounding tissue. To identify bladder cancer tumour‐cell phenotypes and compare classification by tumour‐cell phenotype with classification by global gene expression analysis, we analysed 307 advanced bladder cancers (cystectomized) both by genome gene expression analysis and by immunohistochemistry with antibodies for 28 proteins. According to systematic analysis of gene and protein expression data, focusing on key molecular processes, we describe five tumour‐cell phenotypes of advanced urothelial carcinoma: urothelial‐like, genomically unstable, basal/SCC‐like, mesenchymal‐like, and small‐cell/neuroendocrine‐like. We provide molecular pathological definitions for each subtype. Tumours expressing urothelial differentiation factors show inconsistent and abnormal protein expression of terminal differentiation markers, suggesting pseudo‐differentiation. Cancers with different tumour‐cell phenotypes may co‐cluster (converge), and cases with identical tumour‐cell phenotypes may cluster apart (diverge), in global mRNA analyses. This divergence/convergence suggests that broad global commonalities related to the invasive process may exist between muscle‐invasive tumours regardless of specific tumour‐cell phenotype. Hence, there is a systematic disagreement in subtype classification determined by global mRNA profiling and by immunohistochemical profiling at the tumour‐cell level. We suggest that a combination of molecular pathology (tumour‐cell phenotype) and global mRNA profiling (context) is required for adequate subtype classification of muscle‐invasive bladder cancer. © 2017 The Authors. *The Journal of Pathology* published by John Wiley & Sons Ltd on behalf of Pathological Society of Great Britain and Ireland.

## Introduction

Global mRNA expression analysis is an efficient method for phenotypic profiling of tumours, and has been used to define molecular subtypes for almost every major tumour type 1, 2, 3. However, a key limitation is that tumours, in most cases, are communities of both tumour and non‐tumour cells. Accordingly, the proportions of cells in such communities may severely affect the global gene expression profile. This problem is particularly pertinent for analysis of advanced invasive tumours, which are known to induce major changes and responses in both the tumour and surrounding tissue. We have benefited from our previous analyses of non‐muscle‐invasive (NMI) tumours in defining molecular subtypes of urothelial carcinoma. NMI tumours grow into the bladder cavity with limited influence from surrounding tissue, making it more likely that global gene expression analyses will capture the nature of the tumour‐cell phenotypes. Extensive analyses of NMI tumour samples led us to the definition of three major molecular subtypes of urothelial carcinoma (UC), i.e. urothelial‐like (Uro) (previously termed urobasal 4), genomically unstable (GU), and basal/squamous cell carcinoma (SCC)‐like, with well‐defined molecular phenotypes and genotypes 5, 6, 7, 8. These subtypes show differential activity of established tumour markers and key regulatory genes: Uro tumours express *FGFR3* and *CCND1*, and frequently show 9p21 (CDKN2A) loss; GU tumours express *FOXM1* but not *KRT5*, and frequently show *RB1* loss; and basal/SCC tumours express *KRT5* and *KRT14*, but not *FOXA1* and *GATA3*. Apart from our efforts, UC gene expression subtypes have been described for muscle‐invasive (MI) cases 9, 10, 11, 12 and for NMI cases 13. For MI tumours, the UNC classification 10 limits classification to two subtypes, luminal and basal, and points to a similarity with breast cancer luminal and basal‐like tumours. The MDA classification 9 also defines a luminal and a basal subtype, but introduces a third group, named TP53‐like, distinguished by a TP53‐related gene expression signature. This classification also points to the clinical utility of molecular classification. The TCGA consortium 11 identifies four groups of tumours defined by consensus clustering, but, apart from comparing the clusters with subtypes defined by others, TCGA limits the efforts to name the clusters I, II, III, and IV. With four classification systems at hand, all based on genome‐wide gene expression analyses, Aine *et al* 14 set out to compare the systems by applying all of them to the TCGA dataset. They concluded that the four systems showed extensive overlap and could be organized in a hierarchical order, with the UNC classification at the top with the lowest resolution, followed by the MDA, TCGA and Lund classifications with the highest resolution. However, owing to the complexity of MI carcinomas, the Lund taxonomy had to be extended to include six categories. In the present investigation, we expand previous studies by analysing advanced UC by both global mRNA profiling and by extensive immunohistochemistry (IHC) investigations to define tumour‐cell phenotypes. The results have major implications for how UC should be understood from a molecular perspective.

## Materials and methods

### Sample selection and RNA isolation

Formalin‐fixed paraffin‐embedded preoperative TUR‐B specimens reviewed by a uropathologist were collected from consecutive patients who underwent radical cystectomy in four hospitals in southern Sweden from 2006 to 2011. None of the included tumours received any treatment prior to sample‐taking (Trans uretral resection of the bladder (TUR‐B)). The histological variants at pathological review are shown in supplementary material, Table S1. The pathological stage and grade based on TUR‐B specimens are given in supplementary material, Table S2. Sufficient tissue for embedding of dual cores (1.0 mm) in tissue microarrays (TMAs) and for extraction of RNA was obtained from 307 TUR‐B specimens. For three cases, single cores were embedded. Positioning of the cores within the TUR‐B specimen was performed by selecting areas with >80% tumour cells morphologically and histologically representative of the total tumour. RNA extraction was performed on 4–10 10‐µm sections from macrodissected tissue areas located as close as possible to the positions of the TMA cores. Sampling and RNA extraction are described in detail Supplementary materials and methods. The included samples had an average total spectrometric RNA yield of 2.5 µg (range 0.4–9.3 µg). Informed consent was obtained from all patients, and the study was approved by the Local Ethical Committee of Lund University, in accordance with the Helsinki Declaration.

### Global gene expression analysis

RNA samples were amplified and labelled by use of the SensationPlus kit (Affymetrix, Santa Clara, CA, USA), and raw data were generated with the human Gene ST 1.0 platform (Affymetrix). Batch effects adjustments were performed with the COMBAT algorithm 15. A complete technical description of data processing is included in supplementary material, Supplementary materials and methods. The resulting dataset included 14 062 genes and 307 samples. Raw and normalized data have been deposited in Gene Expression Omnibus under GSE83586. Sample clusters were identified by sequential two‐way splits with the ConsensusClusterPlus R package on the top 50% genes (*n* = 7031) with respect to variance.

### IHC

Antibodies against CCNB1, CCND1, CDH1, CDH3, CDKN2A (p16), CHGA, E2F3, EPCAM, FGFR3, FOXA1, GATA3, KRT5, KRT14, KRT20, NCAM1, PPARG, RB1, RXRA, SYP, TP63, TUBB2B, UPK3, VIM and ZEB2 and antibodies for eight additional supportive markers were used (Supplementary materials and methods). The full dataset (IHC evaluations) is available as supplementary material, Table S6. Staining was also performed with haematoxylin and eosin and with antibodies against CD3, CD68 and smooth muscle actin (ACTA2), to facilitate the identification of immune cell infiltration and stroma. Staining procedures have been described previously 8. Slides were scanned (AxioScan Z1; Zeiss, Oberkochen, Germany), and image files were evaluated with the digital pathology platform PathXL (PathXL, Belfast, UK).

### IHC score calculations and application of IHC subtype definitions

A genomic circuit score 5 was calculated from log2 mRNA expression data and from IHC data as FGFR3 + CCND1 + RB1 – E2F3 of either log2 values (mRNA) or labelling intensity (IHC). Similarly, consensus basal/SCC‐like marker scores (Ba/Sq scores) 4 were calculated as FOXA1 + GATA3 – KRT5 – KRT14 of either log2 values (mRNA) or labelling intensity (IHC). The proposed definitions of tumour‐cell phenotypes were translated to IHC phenotype scores as described in Supplementary materials and methods. Cases with missing data for the defining markers were not classified.

### Statistical analyses

Statistical tests were performed with the R software environment for statistical computing. For group comparisons of IHC data, two‐sided non‐parametric test with significance threshold *α* = 0.05 were used unless otherwise indicated. For group comparisons of gene expression data, false discovery rates were controlled at a maximum of *q* = 0.01.

## Results

### Global gene expression recapitulates previously identified molecular subtypes

**Figure 1:**
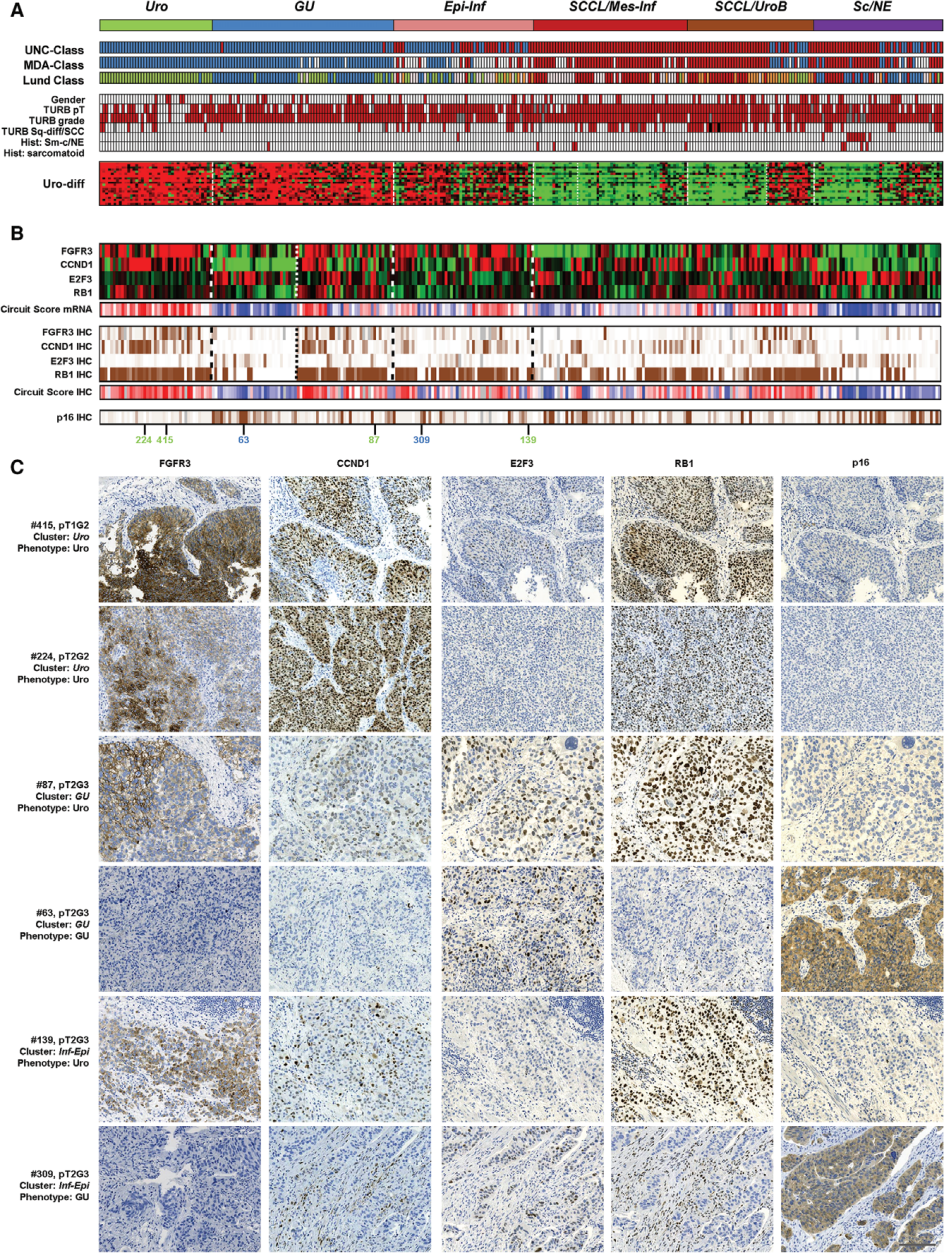
Consensus clustering and Uro‐diff‐positive tumours. (A) The identification of tumours with a dominating luminal character. Consensus clusters obtained by unsupervised clustering of mRNA expression data are shown at the top in relation to classifications obtained with the UNC, MDA and Lund algorithms, as well as clinical and pathological variables. The Uro‐diff signature heatmap shows that the majority of the Uro‐diff‐positive cases are of either the Uro, GU or Epi‐Inf gene expression subtypes. UNC class: blue, luminal; dark red, basal. MDA class: blue, luminal; white, TP53‐like; dark red, basal. Lund class: green, Uro; blue, GU; light brown, UroB; dark red, SCC‐like; white, infiltrated. Red colour indicates female gender, pathological stage ≥T2, pathological grade 3, signs of squamous differentiation, the presence of small‐cell/neuroendocrine histology, and the presence of sarcomatoid histology. Black boxes indicate two cases with pure SCC of the bladder; grey boxes indicate grey boxes indicate grade not determined. (B) Uro and GU phenotypes of the Uro‐diff‐positive subset are identified by the genomic circuit genes FGFR3, CCND1, RB1 and E2F3 at both mRNA level and the IHC level. For completeness, results are given for the whole dataset. Circuit scores were calculated by adding values for FGFR3, CCND1 and RB1, and subtracting that for E2F3, and are depicted in red (high, indicating Uro phenotype) and blue (low, indicating a GU phenotype). IHC scores were percentile‐mapped to a brown (high) and white (low) colour scale. CDKN2A (p16) is also given as an alternative IHC marker in the genomic circuit. Grey bars indicate missing data. Green numbers indicate cases with a Uro phenotype, and blue numbers indicate cases with a GU phenotype in (C). (C) Representative marker profiles of the Uro and GU tumour‐cell phenotypes in the Uro, GU and Epi‐Inf consensus clusters. Each row corresponds to one tumour for which case number (mapping to numbers in Figure 1B), pathological stage and grade, consensus cluster (italics) and tumour‐cell phenotype are given. Each column of images shows staining with the indicated marker. Four cases have identical Uro phenotypes (FGFR3^+^, CCND1^+^, RB1^+^, E2F3^−^, and p16^−^) regardless of stage (pT1 or higher) and consensus cluster (Uro, GU, and Epi‐Inf). Two cases shown have the opposite GU phenotype (FGFR3^−^, CCND1^−^, RB1^−^, E2F3^+^, and p16^+^) found in the GU or in the Epi‐Inf cluster. Scale bar: 100 µm.

We performed stepwise unsupervised hierarchical clustering using whole genome mRNA expression for 307 advanced bladder cancers, resulting in six consensus clusters of approximately equal sizes. To compare the clustering results with known molecular subtypes of UC, we used six gene expression signatures with subtype‐specific expression (supplementary material, Figure S1). On the basis of these signatures, we concluded that the structure of the present data conforms to, and reproduces, clusters obtained in the TCGA RNA‐Seq dataset (*N* = 238) reported by Aine *et al* 14, and assigned the six consensus clusters the following labels: *urothelial‐like* (*Uro*, *n* = 41), *genomically unstable* (*GU*, *n* = 66), *epithelial‐infiltrated* (*Epi‐Inf*, *n* = 51), *Squamous cell carcinoma-like/mesenchymal-infiltrated* (*SCCL/Mes‐Inf*, *n* = 56), *SCCL/UroB* (*n* = 46), and *small‐cell/neuroendocrine‐like* (*Sc/NE*, *n* = 47) (Figure 1A). Throughout the analyses, we will use subtype abbreviations in italics to refer to these gene expression clusters (e.g. *Epi‐Inf)*, whereas we will use non‐italicized abbreviations for tumour‐cell phenotypes determined by IHC (e.g. basal/SCC‐like).

### Tumours with expression of the urothelial differentiation signature (Uro‐diff) have Uro or GU tumour‐cell phenotypes

We compared the obtained clusters with published molecular subtype classification algorithms, clinical and pathological data, and expression of Uro‐diff. The combined data indicate that high Uro‐diff expression is a hallmark of the ‘luminal‐type’ mRNA clusters *Uro*, *GU*, and *Epi‐Inf*, whereas lack of signature expression is characteristic for *SCCL/Mes-Inf*, *SCCL/UroB*, and *Sc/NE* clusters (Figure 1A). Next, we compared global (mRNA) and tumour‐cell specific (IHC) phenotypes of the Uro‐Diff‐positive mRNA clusters *Uro*, *GU*, and *Epi‐Inf*. We used mRNA and protein expression data for FGFR3, CCND1, E2F3, and RB1, which make up a genomic circuit that separates the Uro subtype from the GU subtype (Figure 1B) 5. The calculated genomic circuit scores were, as expected, high for tumours in the *Uro* cluster, i.e. FGFR3^+^, CCND1^+^, RB1^+^, and E2F3^−^, both at the mRNA level and the tumour‐cell protein level (Figure 1B). This was true regardless of stage, as indicated by the T1 tumours included in the study (Figure 1A). In the *GU* consensus cluster, approximately half showed the anticipated strong decrease in circuit score, i.e. FGFR3^−^, CCND1^−^, RB1^−^, and E2F3^+^, whereas the other half showed scores similar to those of the *Uro* cluster. This is in line with results obtained by the Lund classification algorithm, which identified several potential Uro cases as a part of the *GU* cluster, and thus *GU*‐Uro cases (Figure 1A). We confirmed this finding by analysis of CDKN2A (p16) protein expression, which was also low in both *Uro* and *GU‐*Uro cases, but high in *GU‐*GU cases (Figure 1B, C), consistent with *CDKN2A* deletions/mutations being frequent in progressed Uro cases 5 and with GU showing frequent overexpression of p16 8. The *GU*‐Uro tumours differed from *Uro* tumours by increased proliferation, immune and extracellular matrix (ECM) mRNA signatures, but protein expression levels of the canonical Uro genes *FGFR3*, *CCND1* and *TP63* were not different in the *Uro* versus *GU*‐Uro groups of tumours (supplementary material, Figure S2). We therefore conclude that tumours of both Uro and GU tumour‐cell phenotypes are observed in the *GU* consensus mRNA cluster. Analysis of the *Epi‐Inf* consensus cluster at the mRNA level is compromised by high levels of infiltrating non‐tumour cells (supplementary material, Figure S1). However, of 47 *Epi‐Inf* cases subjected to IHC analysis, 30 were considered to have Uro and 10 GU tumour phenotypes, whereas seven had indecisive results at this stage (Figure 1B, C). Thus, the *Epi‐Inf* cluster is mainly composed of tumours with Uro or GU tumour‐cell phenotypes.

### Tumours lacking expression of urothelial differentiation genes have SCC‐like, mesenchymal‐like or neuroendocrine‐like phenotypes

**Figure 2:**
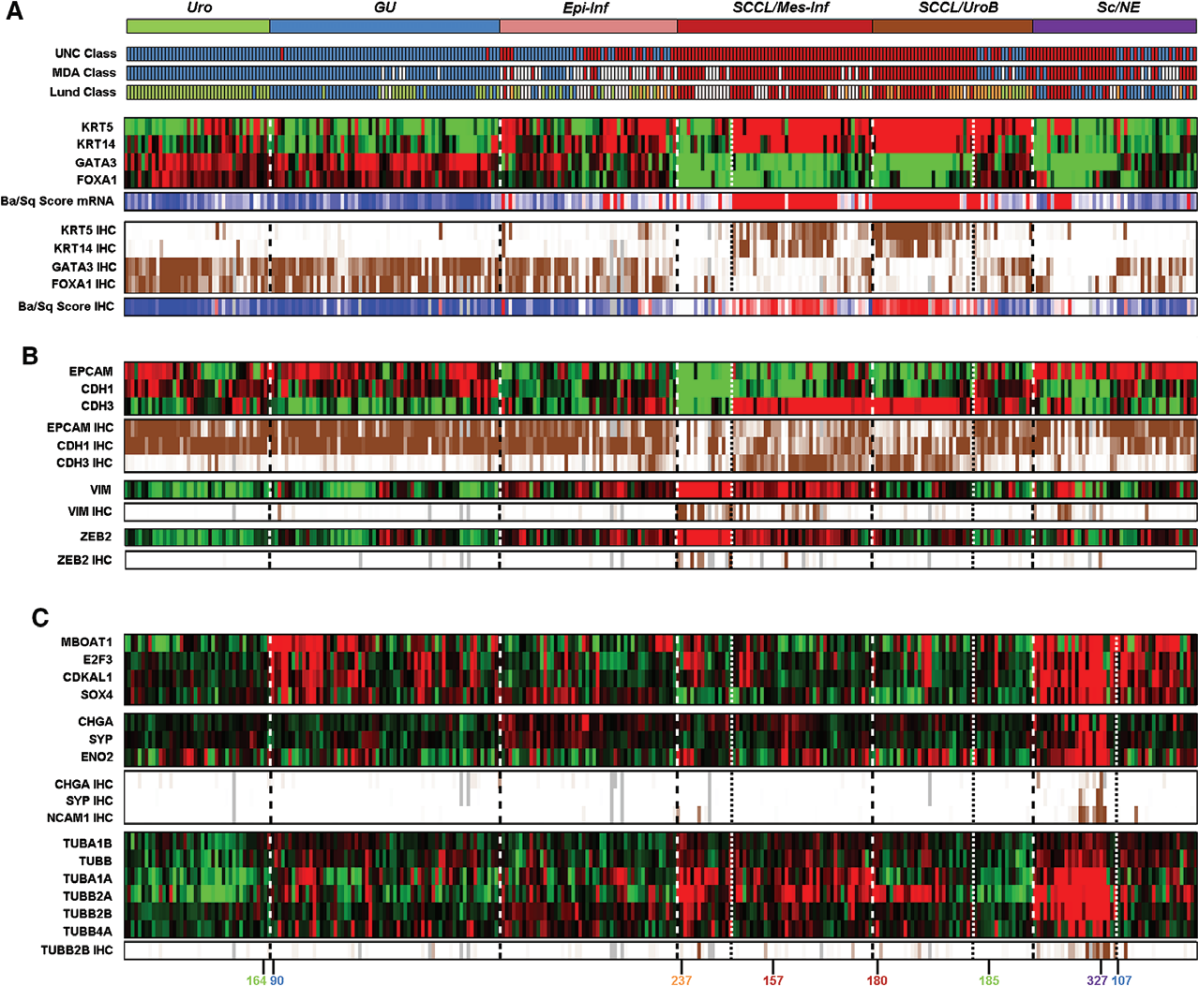
Uro‐diff‐negative tumours are of basal/SCC‐like, mesenchymal‐like or small‐cell/neuroendocrine‐like subtypes. (A) Identification of tumours with a basal/SCC‐like character. Consensus clusters obtained by unsupervised clustering of mRNA expression data are shown at the top in relation to classifications obtained with the UNC, MDA and Lund algorithms. Basal/SCC‐like tumours show high KRT5 and KRT14 expression, and low FOXA1 and GATA3 expression. This tumour‐cell phenotype was observed in both the SCCL/Mes‐Inf and the SCCL/UroB gene expression clusters. Individual markers and a ratio score (Ba/Sq score: red, high score and basal/SCC‐like; blue, low score and non‐basal/SCC‐like) are shown for mRNA and IHC levels. Note the KRT5^−^, KRT14^−^, GATA3^−^ and FOXA1^−^ small non‐basal/SCC‐like group included in the SCCL/Mes‐Inf gene expression consensus cluster. Heatmaps of gene expression are depicted in red (high) and green (low). IHC scores were percentile‐mapped to a brown (high) and white (low) colour scale. Dotted lines separate consensus tumour clusters, and fine dotted lines separate the Mes‐like, UroB and Sc/NE‐like subclusters. (B) Basal/SCC‐like cases express low levels of CDH1 and EPCAM, but high levels of CDH3, at both the mRNA level and the protein level. The KRT5^−^, KRT14^−^, GATA3^−^ and FOXA1^−^ group included in the SCCL/Mes‐Inf gene expression consensus cluster is distinct from the basal/SCC‐like cases in the same cluster by being negative for CDH1, EPCAM, and CDH3. The absence of all three, combined with positivity for VIM and ZEB2 at both the mRNA level and IHC level, identifies a subset of tumours with a mesenchymal‐like phenotype. (C) Dissecting tumour‐cell phenotypes in the Sc/NE consensus cluster. Almost all cases in the Sc/NE gene expression cluster show overexpression of the 6p22 amplicon genes MBOAT1, E2F3, CDKAL1, and SOX4. In contrast, only half of the cluster shows gene and protein expression of the small‐cell/neuroendocrine markers CHGA, SYP, ENO2, and NCAM1. In addition, the same set of tumours was found to have high mRNA expression of several tubulin isoforms (including tubulin β2B, for which IHC data are also shown). Numbers colour‐coded by phenotype indicate the cases for which IHC images are shown in Figures 3 and 4.

**Figure 3:**
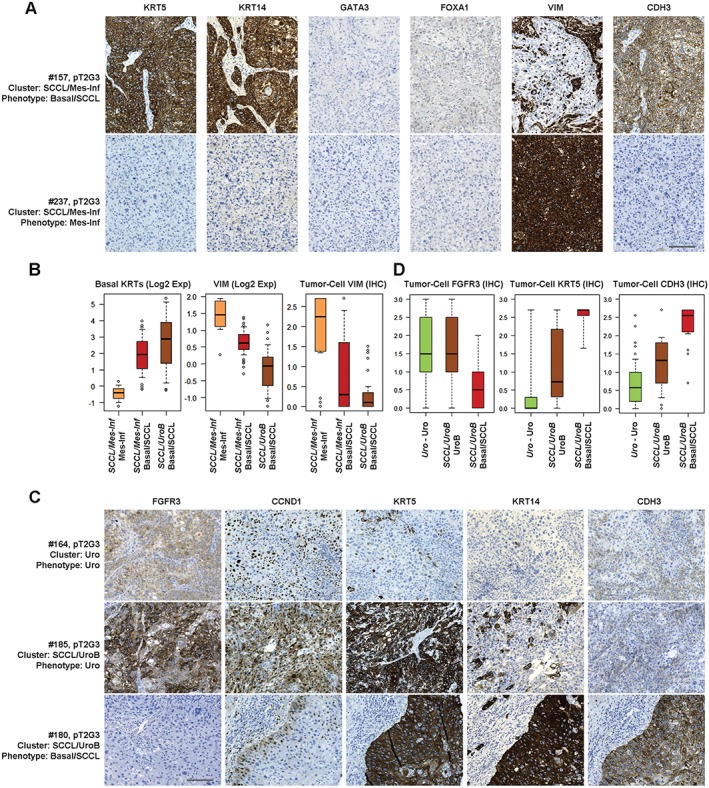
Minor subtypes co‐clustering with basal/SCCL tumours. (A) Representative marker profiles of the basal/SCC‐like and mesenchymal‐like tumour‐cell phenotypes in the SCCL/Mes‐Inf consensus cluster. Each row corresponds to one tumour for which case number (mapping to numbers in Figure 2C), pathological stage and grade, consensus cluster (italics) and tumour‐cell phenotype are given. Each column shows staining with the indicated marker. Despite clustering together, the basal/SCC‐like tumour cells (KRT5^+^, KRT14^+^, CDH3^+^, GATA3^−^, FOXA1^−^, and VIM^−^) are clearly different in type from the mesenchymal‐infiltrated tumours, which are VIM^+^. Both phenotypes lack the urothelial differentiation markers GATA3 and FOXA1. Scale bar: 100 µm. (B) Box plots showing basal keratin (KRT4, KRT5, KRT6A, KRT6B, and KRT14) and VIM mRNA, and tumour‐cell VIM expression (IHC), in the Mes‐Inf subcluster as compared with basal/SCC‐like cases from the same cluster (SCCL/Mes‐Inf) or from the SCC‐like/UroB consensus cluster. (C) Representative marker profiles of the Uro (top), UroB (middle) and basal/SCC‐like (bottom) tumour‐cell phenotypes in the Uro and SCCL/UroB consensus clusters. Each row corresponds to one case for which case number (mapping to numbers in Figure 2C), pathological stage and grade, consensus cluster (italics) and tumour‐cell phenotype are given. Each column shows staining with the indicated marker. By global mRNA clustering, UroB tumours co‐cluster with basal/SCC‐like tumours. Phenotypic analysis shows that, although UroB tumours show more widespread expression of KRT5, KRT14 and CDH3 (P‐cadherin) than Uro tumours, they maintain expression of the typical Uro markers FGFR3 and CCND1. Scale bar: 100 µm. (D) Box plots showing tumour‐cell expression of FGFR3, KRT5 and CDH3 in Uro‐Uro, UroB and basal/SCC‐like samples. Note: the identical high expression of FGFR3 in Uro and UroB tumours, and the absence of expression in basal/SCC‐like tumours; the low KRT5 expression in Uro tumours, the highly variable expression in UroB tumours, and the high expression in basal/SCC‐like tumours; and the low CDH3 (P‐cadherin) expression in Uro tumours, intermediate expression in UroB tumours, and high expression in basal/SCC‐like tumours.

Next, we set out to dissect tumour‐cell phenotypes in the Uro‐diff‐negative subtypes. We used the consensus definition of basal/SCC‐like tumours, KRT5/KRT14‐high and FOXA1/GATA3‐low 4. This set of markers clearly identified the *SCCL/Mes‐Inf* and the *SCCL/UroB* clusters as being composed mainly of basal/SCC‐like cases, with scores based on either mRNA expression or tumour‐cell protein expression (Figure 2A). The typical basal/SCC‐like cases also showed a shift from high EPCAM and CDH1 and low CDH3 expression in *Uro* and *GU* cases to lower EPCAM and CDH1 and high CDH3 expression (Figure 2B). One portion of the *SCCL/Mes‐Inf* cluster was negative for KRT5/KRT14 and FOXA1/GATA3, as well as for CDH3 expression, making it distinct from basal/SCC‐like tumours (Figure 2A, B). The most upregulated mRNAs in this group, as compared with the basal/SCC‐like cases in the same cluster, were *ZEB2* and *VIM* (supplementary material, Table S1), identifying this group as the *Mes‐Inf* mRNA cluster 14. The *Mes‐Inf* tumours were negative for a large number of basal cell‐related and SCC‐related cytokeratins, but positive for tumour‐cell expression (IHC) of both ZEB2 and VIM (Figure 3A, B). It is of note that, at the mRNA level, a large proportion of the basal/SCC‐like tumours in the same consensus cluster expressed *VIM*, but the protein was expressed in infiltrating mesenchymal cells and not by the tumour cells in these cases (Figures 2B and 3A, B). This makes the *Mes‐Inf* tumour‐cell phenotype distinct from the basal/SCC‐like cases and more similar to a mesenchymal than to a basal epithelial phenotype, even though they belong to the same global mRNA‐based tumour cluster.

A subset of tumours in the *SCCL/UroB* cluster showed simultaneous expression of the Uro‐diff, FGFR3 and SCC signatures; although the latter did not reach the levels typically seen for basal/SCC‐like cases (supplementary material, Figure S1). In addition, the same cases were classified as UroA or UroB by the Lund classifier (Figure 2A). Tumours in this subset expressed FGFR3 and CCND1 as determined by IHC at the same levels as the *Uro* tumours, indicating a Uro‐related phenotype. Hence, this portion of the consensus cluster corresponds to UroB tumours. The basal/SCC‐like cases in the same consensus cluster showed almost no expression of FGFR3 and CCND1, whereas the reverse was seen for KRT5 and CDH3 tumour‐cell expression (Figure 3C, D). A significance analysis of microarrays (SAM) on mRNA data clearly distinguished the UroB portion from the basal/SCC‐like portion in the same cluster, with the canonical Uro‐diff genes *GATA3*, *FOXA1* and *PPARG* being among the top upregulated genes (supplementary material, Table S3). Conversely, the phenotypically basal/SCC‐like cases were distinguished by expression of SCC/desmosome markers, e.g. *KRT6B* and *DSC2*. Taken together, these findings indicate that the UroB tumour‐cell phenotype is distinct and more similar to a Uro phenotype than to a basal/SCC‐like phenotype, even though they are grouped within the same cluster by global mRNA clustering.

The above results define two possible basal/SCC‐like categories, one with strong ECM and T‐cell signatures that segregates with the Mes‐Inf subtype, and one with weaker ECM and T‐cell signatures that segregates with the UroB subtype, on global mRNA clustering. However, these two basal/SCC‐like tumour categories do not differ in their KRT5, KRT14/FOXA1, GATA3 ratios, their defining characteristics, or their shifts to high CDH3 and lower CDH1 expression. Hence, the difference between these two categories, and the reason for them to be part of two different global mRNA clusters, is determined by signatures related to infiltrating non‐tumour cells.

**Figure 4:**
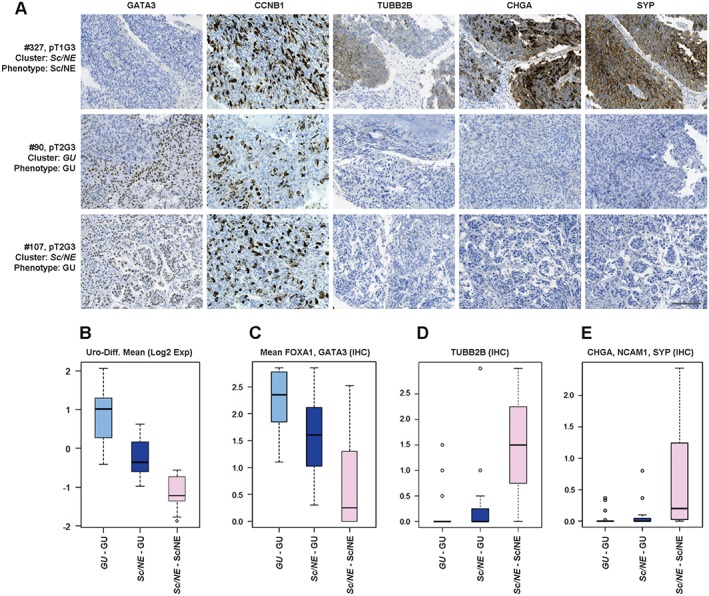
The Sc/NE consensus cluster is composed of tumours with Sc/NE‐like and GU tumour‐cell phenotypes. (A) Representative marker profiles of the Sc/NE and GU tumour‐cell phenotypes in the Sc/NE and GU consensus clusters. Each row corresponds to one tumour, with case number (mapping to numbers in Figure 2C), pathological stage and grade, consensus cluster (italics) and tumour‐cell phenotype given. Each column shows staining with the indicated marker. An IHC profile of a tumour from the Sc/NE consensus cluster (top row) shows a typical Sc/NE‐like profile; negative for GATA3, and positive for CCNB1, TUBB2B, CHGA, and SYP. A tumour with a typical GU phenotype from the GU consensus cluster (middle row) is positive for GATA3 and CCNB1, and negative for TUBB2B, CHGA and SYP. A tumour from the Sc/NE consensus cluster shows a typical GU tumour‐cell phenotype (bottom row). Scale bar: 100 µm. (B–E) Boxplots showing mean mRNA expression of (B) the Uro‐diff signature, (C) mean protein tumour‐cell expression of GATA3/FOXA1, (D) TUBB2B expression and (E) mean protein expression of CHGA/NCAM1/SYP in cases with a GU tumour‐cell phenotype from the GU consensus cluster, cases with a GU tumour‐cell phenotype from the Sc/NE cluster, and cases with an Sc/NE‐like tumour‐cell phenotype from the Sc/NE consensus cluster, respectively.

Cases in the *Sc/NE* consensus cluster showed coordinated overexpression of *E2F3*, *CDKAL1*, *SOX4* and *MBOAT1*, which are genes located in the core region of the 6p22 amplicon (Figure 2C). The *Sc/NE* tumours share this feature with those showing a true GU IHC phenotype in the *GU* cluster. To resolve the *Sc/NE* cluster further, we used mRNA levels for *CHGA*, *SYP* and *ENO2* and applied IHC for CHGA, SYP, and NCAM1 (CD56), all of which are markers for either a small cell or a neuroendocrine phenotype 16 (Figure 2C). Only one‐half of the *Sc/NE* consensus cluster showed enriched expression of the markers. SAM revealed that several tubulin genes were among the top genes co‐expressed with markers for a neuroendocrine phenotype (supplementary material, Table S3). The top upregulated genes were normally restricted to cells of neuroendocrine origin (supplementary material, Figure S3). Expression of *TUBB2B* (tubulin β2B), the second most significant upregulated gene in this analysis, coincided with expression of the small‐cell/neuroendocrine differentiation genes (Figure 2C), and IHC showed that it was expressed by tumour cells (Figure 4A). This identifies a group of urothelial carcinomas with a tumour‐cell phenotype reminiscent of neuroendocrine cells. We named this phenotype small‐cell/neuroendocrine‐like (Sc/NE‐like). However, these features accounted for only one half of the *Sc/NE* consensus cluster. The second half showed significantly higher mRNA expression levels of the Uro‐diff gene signature (Figure 4B; supplementary material, Figure S4), as well as of the key transcription factors FOXA1 and GATA3 (Figure 4C; supplementary material, Figure S4), and appeared to be indistinguishable from GU cases at the tumour‐cell level. Expression of canonical neuroendocrine markers was not detected in this half of the cluster (Figure 4D, E). SAM confirmed that the Uro‐diff signature drives the separation of these subclusters, as the canonical genes *GATA3*, *UPK2* and *PPARG* were among the top 10 genes as compared with Sc/NE‐like cases (supplementary material, Table S3). Hence, this group has several features in common with the Uro‐diff‐positive half of the cohort, and with a GU tumour‐cell phenotype in particular.

### Pseudo‐differentiation in advanced UC with Uro and GU phenotypes

**Figure 5:**
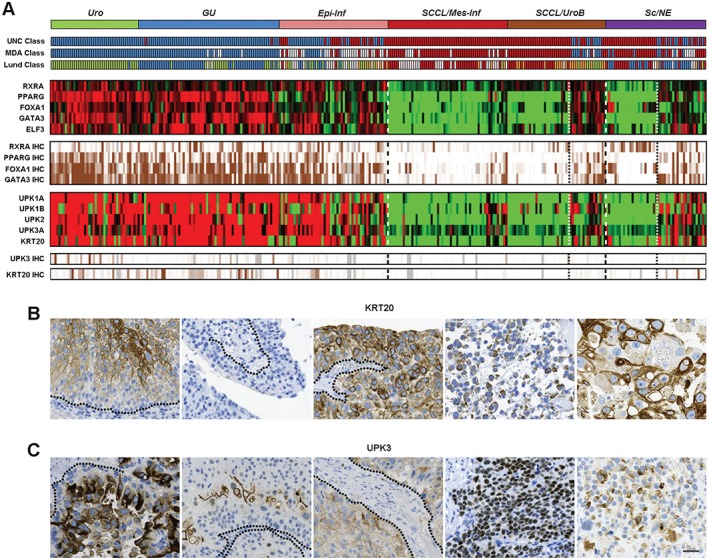
The normal terminal differentiation markers KRT20 and UPK3 are inconsistently and aberrantly expressed in Uro‐diff‐positive tumours, indicating pseudo‐differentiation. (A) The identification of tumours that express differentiation‐related transcription factors and differentiation readout markers. Consensus clusters obtained by unsupervised clustering of mRNA expression data are shown in relation to classifications obtained with the UNC, MDA and Lund classification algorithms. Expression (mRNA) of the indicated transcription factor genes and tumour‐cell protein expression determined by IHC are shown as heatmaps. Note the expression of urothelial regulatory factors in both UroB cases and GU cases in the Sc/NE consensus cluster. Similarly, expression (mRNA) of the terminal differentiation marker genes UPK1A, UPK1B, UPK2, UPK3A and KRT20, and tumour‐cell protein expression of UPK3 and KRT20, are shown. Heatmaps of gene expression are depicted in red (high), and green (low). IHC scores were percentile‐mapped to a brown (high) and white (low) colour scale. Dotted lines indicate the consensus cluster borders between Uro‐diff‐positive and Uro‐diff‐negative clusters and subclusters. (B) Examples of various forms of aberrant KRT20 protein expression. Each image represents an individual tumour. From the left: staining of intermediate cells; no staining of any cell layer; staining of all cell layers; staining of loosely attached cells with a very low cellular differentiation level; staining of pleomorphic, large cells with a low cellular differentiation level. (C) Examples of aberrant UPK3 protein expression. Each image represents an individual tumour. From the left: staining of intermediate cells; staining only of cells most distal to the tumour–stroma interface; staining of all cell layers; strong nuclear staining; cytoplasmic staining of cells with a low cellular differentiation level. In (B) and (C), dotted lines indicate basal membrane. Scale bar: 20 µm.

We then inspected the expression of the transcription factor genes *RXRA*, *PPARG*, *FOXA1*, *GATA3*, and *ELF3*, which are known to be central in differentiation of the normal urothelium 17, 18, 19, 20, 21. These factors showed coordinated high expression in both the *Uro* and *GU* and in the *Epi‐Inf* consensus clusters, as determined by both mRNA analysis and IHC (ELF3 was not included in the latter analysis) (Figure 5A). Coordinated downregulation of these genes was observed in the *SCCL/Mes‐Inf* and *SCCL/UroB* clusters. In the latter group, UroB cases showed expression of these factors to a varying extent. The *Sc/NE* consensus cluster was split into two groups, one associated with the Sc/NE‐like cases with an absence of factor expression, and one in which the factors and signature were expressed at varying levels, further strengthening the conclusion that the *Sc/Ne* consensus cluster consists of tumours of two different tumour‐cell phenotypes.

Next, we investigated the relationship between relative mRNA levels and the tumour‐cell expression of the terminal differentiation markers KRT20 and UPK3. We hypothesized that not only marker levels but also subcellular expression patterns may reveal where and if urothelial differentiation takes place. KRT20 and UPK3 staining was remarkably inconsistent; a multitude of aberrant and heterogeneous expression patterns were observed for each marker, and, unlike that of the regulatory factors, KRT20 and UPK3 expression was hardly ever homogeneous in a tumour area 1 mm in diameter. Among the most conspicuous of these observations was the stochastic appearance of tumour‐cell KRT20 staining, and, even more, the aberrant subcellular localization of these markers (Figure 5B, C). Moreover, KRT20 and UPK3 were observed in each of the three types of invasive growth pattern described for UC, i.e. infiltrative, nodular, and trabecular 22 (supplementary material, Figure S5), as well as in cells with low differentiation levels, loss of cellular adhesion, and highly atypical and pleomorphic nuclei (Figure 5B, C; supplementary material, Figures S5 and S6). Taken together, these results show that regulatory factors accurately identify Uro‐diff‐positive tumours at both the mRNA level and the protein level, but, whereas *KRT20* and *UPK3* mRNA expression overlaps with the Uro‐diff signature, only subsets of the tumour cells are positive at the IHC level, and expression patterns are inconsistent with the proteins' structural functions in normal differentiation.

### Tumour‐cell phenotype definitions

**Figure 6:**
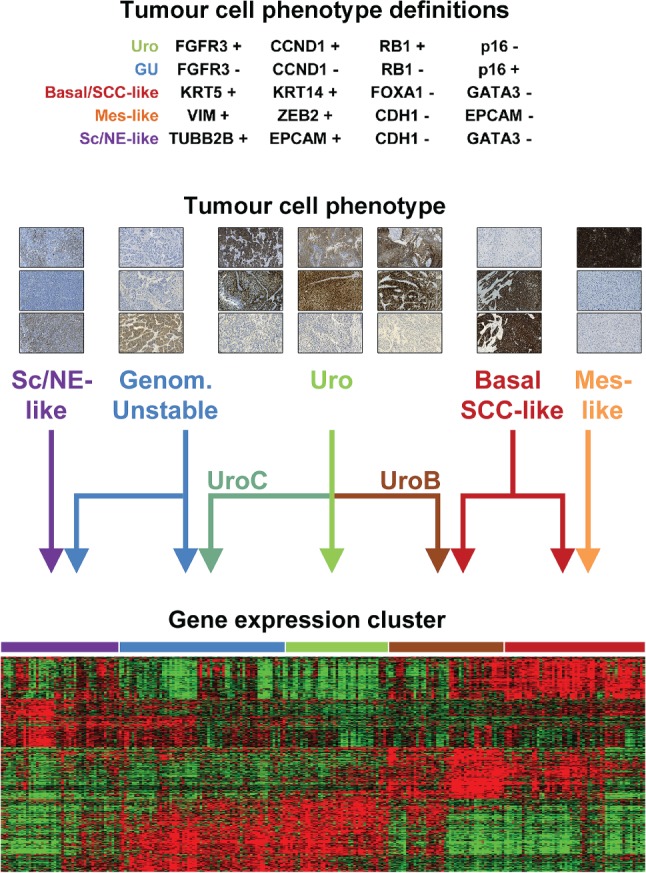
Tumour‐cell phenotype definitions and tumour‐cell phenotype relationships with gene expression clusters. IHC definitions of the urothelial‐like (Uro), genomically unstable (GU), basal/SCC‐like (SCC‐like), mesenchymal‐like (Mes‐like) and small‐cell/neuroendocrine‐like (Sc/NE‐like) phenotypes (see supplementary material for details). Example IHC images (from the top): Sc/NE‐like phenotype – TUBB2B, CDH1, and NCAM1; GU phenotype – FGFR3, CCND1, and CDKN2A (p16); Uro phenotype from cases in the three different clusters indicated by the arrows – FGFR3, CCND1, and CDKN2A (p16); Basal/SCC‐like phenotype – GATA3, KRT5, and KRT14; Mes‐like phenotype – VIM, EPCAM, and E‐cadherin. The heatmap shows the top 100 genes from each group (group mean) in a five‐class anova based on consensus clusters. Clusters were re‐ordered to correspond approximately to arrow positions. Gene order was determined by hierarchical clustering. The Epi‐Inf consensus cluster is omitted from the figure.

On the basis of the extensive IHC analyses, we propose working definitions for five major tumour‐cell phenotypes in advanced bladder cancer: urothelial‐like (Uro), genomically unstable (GU), basal/SCC‐like, mesenchymal‐like (Mes‐like), and small‐cell/neuroendocrine‐like (Sc/NE‐like), as described in Figure 6. As there is no reliable way to distinguish UroA from UroB, or from *GU*‐Uro, these tumours are grouped under the ‘Uro’ heading. The defining protein markers represent key genes in tumour biology central to the respective tumour cell phenotypes. We then assigned each tumour an mRNA phenotype based on the deconstructed consensus clusters, and grouped *Uro*‐Uro with *GU*‐Uro and *SCCL/UroB*‐UroB in one Uro class, combined *GU*‐GU and *Sc/NE*‐GU into one GU class, and combined all basal/SCC‐like cases into one class, and tested to what extent the IHC tumour phenotype definitions could recreate the data (supplementary material, Figures S7 and S8). The overall accuracy was 75%, being mainly reduced by the low sensitivity for the two small Mes‐like and Sc/NE‐like classes of tumour. The sensitivity was, however, high for Uro and GU tumours (0.89 and 0.79, respectively), and the specificity for the basal/SCC‐like tumour‐cell phenotype definition was excellent (0.93) (supplementary material, Tables S4 and S5). Hence, we see the presented tumour phenotype definitions as starting points for efficient classification of advanced bladder cancer into relevant and distinct molecular tumour‐cell phenotypes.

## Discussion

Recent advances in the field of UC classification have put increasing weight on urothelial differentiation signatures to subdivide UCs. Such a signature is the driving force behind both the UNC and the MDA classes termed ‘luminal’ 9, 10. With the nomenclature used in the present investigation, their ‘luminal’ categories consist of our *Uro*, *GU* and *Epi-Inf* clusters of gene expression. Indeed, our urothelial differentiation mRNA signature (Uro‐diff) includes both *KRT20* and uroplakin genes normally expressed in luminal cells. Furthermore, the luminal category of tumours frequently express the transcription factors RXRA, PPARG, FOXA1, and GATA3, which are known to have a crucial role in urothelial differentiation. However, the full complement of factors is not always expressed in tumour cells (Figure 5A) 23. We compared gene expression data and IHC data for the terminal differentiation markers KRT20/UPK3, and for RXRA, PPARG, FOXA1, and GATA3, to establish to what extent urothelial differentiation occurs in the Uro‐diff signature subtypes. According to the mRNA data, a large proportion of the tumours expressed both *KRT20* and *UPK3*. Protein expression was, however, typically absent or patterns were far from normal. This is in contrast to NMI cases, which may retain KRT20 and UPK expression in the most apical cells of the tumour papillae 24, 25. We consequently hypothesize that the urothelial differentiation programme becomes corrupted during progression, that KRT20 and uroplakins lose their relevance as luminal differentiation markers, and that pseudo‐differentiation occurs in advanced urothelial tumours.

By exploring gene expression signatures, tumour clustering, and data on tumour‐cell expression of a large panel of IHC markers, we arrived at definitions of five tumour‐cell phenotypes: Uro, GU, basal/SCC‐like, Mes‐like, and Sc/NE‐like. We see these as working definitions that have to be validated in independent series of tumours for which both global mRNA and IHC data are available. The definition of the Uro category of tumours does not distinguish between the predominantly NMI UroA, and UroB. Furthermore, a large proportion of the invasive cases with a Uro tumour‐cell phenotype clustered with GU cases in the *GU* consensus cluster, making them different from both UroA and UroB. We tentatively name this group of Uro tumours UroC. This group differs from early‐stage UroA by being invariably high‐grade, by having lost urothelial‐like stratification, and by showing frequent *CDKN2A* genomic losses 14. UroC shares this last feature with UroB, but, whereas UroB cases start to attain SCC‐like features and cluster together with frank basal/SCC‐like cases, UroC cases instead become increasingly similar to, and finally converge with, frank GU cases at the global gene expression level. As UroA, UroB and UroC are very similar in fundamental gene regulatory features, and thus in ‘type’, designing a simple IHC marker system to distinguish them as separate entities is challenging. Information on global gene expression profiles (cluster assignment) would, in this case, be useful. Nevertheless, this makes Uro the most heterogeneous of the molecular phenotypes and with the most prominent signs of biological progression routes.

The definition of basal/SCC‐like urothelial tumours is established and conforms well to definitions of similar subtypes in other tumour types, i.e. basal‐like breast cancer and SCC of the lung. Nevertheless, frank basal/SCC‐like UC was grouped into two different global gene expression clusters. In this case, divergence is most likely attributable to the profiles of tumour‐infiltrating non‐tumour cells. One cluster, *SCCL/Mes‐Inf*, showed a strong ECM signature, indicating the presence of a large proportion of mesenchymal cells in these tumours. On application of the tumour‐cell phenotype approach, it became evident that a portion of these tumours did not conform to the basal/SCC‐like definition. Instead, the tumour cells were themselves mesenchymal‐like and expressed the typical mesenchymal genes *ZEB2* and *VIM*, indicating a tumour type that has undergone epithelial–mesenchymal transition (EMT). The basal/SCC‐like tumour samples also expressed *ZEB2* and *VIM*, but only in surrounding stromal cells. Thus, the Mes‐like subtype defines a new entity of bladder cancer showing a tumour‐cell phenotype that is in stark contrast to previously defined subtypes, and cases of which are biologically very different from the basal/SCC‐like cases that they cluster together with.

The consensus cluster *Sc/NE‐like* turned out to harbour two very distinct tumour‐cell phenotypes. One‐half of these tumours expressed markers that are typical for neuroendocrine differentiation, e.g. SYP and ENO2, and, more robustly, TUBB2B. This and other tubulin isoforms have normal expression that is highly specific for tissues of neuroendocrine origin. This part of the *Sc/NE* consensus cluster also showed an absence of PPARG, FOXA1 and GATA3 expression, as well as of uroplakin and KRT20 expression. We name this tumour‐cell phenotype small‐cell/neuroendocrine like (Sc/NE‐like). The second half of the *Sc/NE* consensus cluster expressed PPARG, FOXA1, GATA3, and ELF3, as well as the Uro‐diff signature. This, in combination with the absence of FGFR3 and CCND1 protein expression, suggests that these tumours have a GU tumour‐cell phenotype. The forces driving Sc/NE‐like and GU tumours into one consensus cluster are most likely a high proliferation rate and frequent *E2F3*, *CDKAL1* and *SOX4* genomic amplification at 6p22, which are characteristic of both subtypes.

The main finding of the present study is that global mRNA clustering and tumour‐cell phenotype analyses lead to different groupings of bladder cancer samples. We observed several instances where global mRNA clusters did not show the expected tumour‐cell phenotype: (1) approximately half of the tumours in the *GU* consensus cluster were of the Uro tumour‐cell phenotype; (2) basal/SCC‐like tumours were allocated to two different global mRNA tumour clusters; (3) the two small but distinct tumour subtypes Mes‐like and UroB grouped with typical basal/SCC‐like cases; (4) the *Epi‐Inf* consensus cluster was found to consist of cases with Uro and GU tumour‐cell phenotypes; and (5) the *Sc/NE* consensus cluster consisted of tumours with typical neuroendocrine and GU tumour‐cell phenotypes. We interpret these observations as signs of convergence/divergence at the global gene expression level that become evident when tumour phenotypes are measured both at the tumour‐cell level and by global mRNA profiling, as shown schematically in Figure 6. Hence, a complex relationship is apparent between global mRNA clusters and tumour‐cell phenotypes in UC. It is worth noting that this effect is only observed in an MI setting, and not in NMI‐based datasets (e.g. 7, 26), suggesting that broad global commonalities, perhaps related to the invasive process itself, exist between MI tumours regardless of subtype. This also makes the denotation of tumour subtype labels problematic; is the label intended to denote a specific tumour‐cell type, or a tumour type with a given composite organization? This discrepancy suggests that a bi‐nominal classification system consisting of both tumour‐cell phenotype and gene expression cluster (context) would be more appropriate. Such a bi‐nominal system may also bridge the gap between genome‐wide expression profiling and traditional (molecular) pathology. Future clinical studies/trials will show when, and under what circumstances, such a high‐resolution classification system is needed, and when a low‐resolution classification is fully adequate.

## Author contributions statement

GS: performed IHC, including selection of antibodies, scoring of staining, and biological interpretation; PE: performed bioinformatic analyses, including hierarchical clustering and specific signature analyses; FL: responsible for data collection, including pathological re‐evaluation, clinical follow‐up, and ethical issues; MH: conceived the study, was responsible for final interpretations and conclusions, and was responsible for the final draft of the manuscript. All authors contributed to the drafting of the manuscript.

**SUPPLEMENTARY MATERIAL ONLINE**

**Supplementary materials and methods**

**Supplementary figure legends**

**Figure S1.** Consensus clustering of global mRNA expression from cystectomized patients (TUR‐B samples)

**Figure S2.** Cases with Uro phenotype that cluster in the *GU* consensus cluster retain their Uro molecular profile, but proliferation, T‐cell and ECM signals are affected

**Figure S3.** Top 10 upregulated genes in *Sc/NE*–Sc/NE group (compared to *Sc/NE*–GU) frequently have a normal tissue expression pattern restricted to neuronal/endocrine tissues

**Figure S4.** Cases with GU phenotype that cluster in the *Sc/NE* consensus cluster retain expression of the Uro‐Diff signature (Mean mRNA) and key factors (IHC)

**Figure S5.** Protein expression pattern of KRT20 in advanced urothelial carcinoma is de‐regulated and inconsistent with normal urothelial differentiation

**Figure S6.** Protein expression of UPK3 in advanced urothelial carcinoma is de‐regulated and inconsistent with normal urothelial differentiation

**Figure S7.** IHC data from 29 markers showing differential expression in tumour cells were considered in establishing tumour‐cell phenotype definitions

**Figure S8.** Concordance of tumour‐cell phenotype with consensus clusters and deconstructed subtypes

**Table S1.** Histological variants included in the consecutive series of 307 tumours according to the 2016 WHO classification of invasive bladder tumours

**Table S2.** The pathological stage and grade based on uro‐pathologist re‐evaluation of TUR‐B specimens for the consecutive series of 307 tumours

**Table S3.** Differentially expressed genes between Mes‐Inf and Basal/SCC‐like, between UroB and Basal/SCC‐like, and between Sc/NE and GU

**Table S4.** Comparison of tumour‐cell phenotypes (applied IHC definitions) to global mRNA consensus clusters (top)

**Table S5.** Comparison of tumour‐cell phenotypes (applied IHC definitions) to deconstructed mRNA clusters (as described in text)

**Table S6.** The complete IHC‐evaluation data set

## Supporting information


**Supplementary Materials and Methods**

**Supplementary figure legends**

**Supplementary Figure S1 Consensus clustering of global mRNA expression from cystectomized patients (TUR‐B samples).** Obtained tumour clusters are indicated on top. Subtype labels are adapted from Aine et al., 2015 15. Subtype classification are according to UNC‐class; blue, luminal; dark red, basal (Damrauer et al., 2014 10). MDA‐class; blue, luminal; white, TP53‐like; dark red, basal (Choi et al., 2014 9). Lund class; green, Uro; blue, GU; light brown, UroB; dark red, SCC‐like; white, infiltrated (Sjödahl et al., 2012 7). Clinical variables. Gender, white, male; dark red, female. TUR‐B pathological stage, dark red, ≥T2; white < T2. TUR‐B pathological grade, dark red, G3; white, <G3; grey, Gx. Squamous differentiation, dark red, sings of squamous differentiation; black, pathological SCC tumours. Small cell or neuroendocrine histology, dark red, presence of Sc/NE histology. Sarcomatoid histology, dark red presence of sarcomatoid histology. Heatmaps of gene expression are depicted in red (high), and green (low), colour scale. Heatmaps for genes signatures correspond to the urothelial differentiation signature (Uro‐diff), genes associated with FGFR3 mutation/overexpression (FGFR3 cluster), late cell cycle (proliferation) signature, genes upregulated in squamous cell carcinoma of the urothelium (SCC vs. UC, Blaveri et al., 2005 [27]), genes expressed by T‐cells (T‐cell), and gene related to extra cellular matrix (ECM). Citation numbers refer to the main text.

**Supplementary Figure S2 Cases with Uro phenotype that cluster in the GU consensus cluster retain their Uro molecular profile but proliferation, T‐cell and ECM signals are affected.** For each plot, the y‐axis label indicates the data type (IHC, or mRNA‐signature). Cases with Uro phenotype in the GU consensus cluster (dark green) are shown to have increased CCNB1 staining (proliferation), T‐cell‐, and ECM‐Signatures, compared to their Uro ‐ Uro counterpart (light green). For canonical Uro markers (FGFR3, CCND1, TP63), however, there was no difference, confirming their molecular profile as Uro tumours. Tumours with a GU phenotype lack expression of these canonical markers.

**Supplementary Figure S3 Top 10 upregulated genes in Sc/NE – Sc/NE group (compared to Sc/NE – GU) frequently have a normal tissue expression pattern restricted to neuronal/endocrine tissues.** SAM analysis was perfomed comparing the two groups and the top 10 upregulated genes were queried in the gTEX database (RNA‐Seq data from normal tissues, gtexportal.org, gTEX consortium, 2015 [28]). Boxplots of FPKM values; Yellow boxes represent brain tissues of different anatomical locations, light green represents pituitary gland, orange represents tibial nerve. Several of the most upregulated genes show normal expression restricted to neuronal/endocrine tissues. Citation numbers refer to the main text.

**Supplementary Figure S4 Cases with GU phenotype that cluster in the Sc/NE consensus cluster retain expression of the UroDiff‐signature (Mean mRNA) and key factors (IHC).** For each plot, the y‐axis label indicates the data type (IHC, or mRNA‐signature). Cases with GU phenotype in the Sc/NE consensus cluster (dark blue) are shown to have retained expression of Uro‐diff signature (left) and tumour cell expression of GATA3, FOXA1, and PPARG. Curiously RXRA (IHC) indicated high levels in the cases with Sc/NE phenotype, which was not the case for the RXRA values in the gene expression data set. Thus, Sc/NE ‐ Sc/NE cases lose urothelial differentiation signature, except for possibly RXRA protein expression, whereas the cases with GU phenotype in the same consensus cluster do not.

**Supplementary Figure S5 Protein expression patterns of KRT20 in advanced urothelial carcinoma is de‐regulated and inconsistent with normal urothelial differentiation.** (A) KRT20 positive strong cytoplasmic staining. (B) KRT20 positive strong membraneous staining. (C) KRT20 positive case where basal cells are negative. All cells distal to the tumour‐stroma interface are positive. (D) KRT20 positive case where few cells most distal to the tumour‐stroma interface are positive. (E) KRT20 positive case where bulk tumour, basal cells, and a lamina propria invading nest are positive. (F) KRT20 positive case with an infiltrative invasive growth pattern. (G) KRT20 positive case with a nodular invasive growth pattern. (H) KRT20 positive case with a trabecular invasive growth pattern. (I) Dysplastic urothelium with highly variable KRT20 staining. (J‐K) KRT20 positive mucinous tumours with signet‐ring morphology. Scale bars indicate 100 µm. (L) Sample including intestinal metaplasia, showing KRT20 positivity. (M) KRT20 positive case with heterogeneous expression (non‐clonal appearance). (N) KRT20 positive case with heterogeneous expression (clonal appearance). (O) KRT20 positive strongly lymphocyte‐infiltrated Genomically Unstable tumour (Sc/NE ‐ GU). (P) CD3 staining of the same case as in (O), showing dense lymphocyte infiltration. Scale bars for (A‐I) and (L‐O) indicate 20 µm.

**Supplementary Figure S6 Protein expression of UPK3 in advanced urothelial carcinoma is de‐regulated and inconsistent with normal urothelial differentiation.** (A) UPK3 positive case showing strong nuclear staining. (B) UPK3 positive case showing cytoplasmic staining. (C) Strong apical UPK3 positivity in luminal cells that lack normal urothelial organization. (D) UPK3 positive case with an infiltrative invasive growth pattern. (E) UPK3 positive case with a nodular invasive growth pattern. (F) UPK3 positive case with a trabecular invasive growth pattern. (G) Tumour nests displaying a ‘pushing‐border’ growth pattern with UPK3 positivity along the nests' circumference, resulting in an inverted staining pattern (cells closest to tumour‐stroma interface positive). (H) UPK3 positive case with heterogeneous expression (non‐clonal appearance). (I) UPK3 positive case with heterogeneous expression (clonal appearance). Scale bars indicate 20 µm.

**Supplementary Figure S7, See also Supplementary Figure 8. IHC data from 29 markers showing differential expression in tumour cells were considered in establishing tumour‐cell phenotype definitions.** Barplots showing the mean IHC scores for the deconstructed gene expression phenotypes including UroB, as we initially intended to produce a separate IHC definition also for UroB. Uro includes Uro cases from the Uro, GU, and Epi‐Inf clusters. GU includes GU cases from the GU, Epi‐Inf, and Sc/NE clusters. Basal/SCCL includes Basal/SCC‐like cases from the SCCL/Mes‐Inf and SCCL/UroB clusters.

**Supplementary Figure S8, Concordance of tumour‐cell phenotype with consensus clusters and deconstructed subtypes.** (A) IHC classification based on definitions in Figure 6 of tumours, grouped by the consensus clusters Urothelial‐like (Uro), Genomically Unstable (GU), Epithelial Infiltrated (Epi‐Inf), SCC‐like/Mesenchymal Infiltrated (SCCL/Mes‐Inf), SCC‐like/Urothelial‐like B (SCCL/UroB), and the Small‐cell/Neuroendocrine (Sc/NE). (B) IHC classification based on definitions in Figure 6 of tumours, grouped according to deconstructed tumour subtypes as determined by examination of mRNA profiles. Uro, composed of Uro cases from the Uro, GU, and Epi‐Inf consensus clusters, and UroB cases from the SCC‐like/UroB consensus cluster. GU, composed of GU cases from the GU, Epi‐Inf and Sc/NE consensus clusters. SCC‐like, composed of Basal/SCC‐like cases from the SCC‐like/Mes‐Inf and SCC‐like/UroB consensus clusters. Mes‐Inf and Sc/NE subclusters as defined in the text.

**Table S1.** Histological variants included in the consecutive series of 307 tumours according to the 2016 WHO classification of invasive bladder tumours.
**Table S2.** The pathological stage and grade based on uro‐pathologist re‐evaluation of TUR‐B specimens for the consecutive series of 307 tumours.
**Table S3.** Differentially expressed genes between Mes‐Inf and Basal/SCC‐like, between UroB and Basal/SCC‐like, and between Sc/NE and GU.
**Table S4.** Comparison of tumour‐cell phenotypes (applied IHC definitions) to global mRNA consensus clusters (top).
**Table S5.** Comparison of tumour‐cell phenotypes (applied IHC definitions) to deconstructed mRNA clusters (as described in text).

**Table S6** The complete IHC‐evaluation data set.
