# Supplementary material for: Molecular classification of urothelial carcinoma: global mRNA classification versus tumour‐cell phenotype classification
Source: J Pathol. 2017 Mar 28;242(1):113–25. doi: 10.1002/path.4886 (PMC5413843; doi:10.1002/path.4886)
Supplement: Supplementary file 1 — Supplementary Materials and Methods [file PATH-242-113-s007.docx]

**Coates edited**

**Supplementary Materials and Methods**

**Sample collection**

From a cohort of patients that underwent radical cystectomy, pathological FFPE-blocks containing tissue from trans-urethral resection of the bladder (TUR-B) were identified by revision of Haematoxylin & Eosin (H&E) stained slides by a uro-pathologist. For included pathological blocks, H&E staining was performed on a fresh whole section which was used to guide sampling. Sampling was done by initial placement of four (2x2) TMA cores in representative tissue areas by a single technician. TMA cores were punched out and placed into two TMAs containing two cores each per sample. The first TMA was used in this study. Next, an awl was used to carve around an area of tumour tissue. This area was placed surrounding the holes left after punching out the TMA cores whenever possible (see example below). When this was not possible the macro-dissected area could be located in a different part of the TUR-B block from the TMA cores. For RNA extraction, four to ten (depending on the size of the area) 10 µm sections were taken and the flakes containing the macro-dissected area were transferred to tubes for immediate deparaffinization.


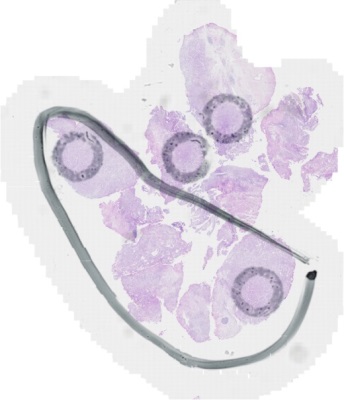

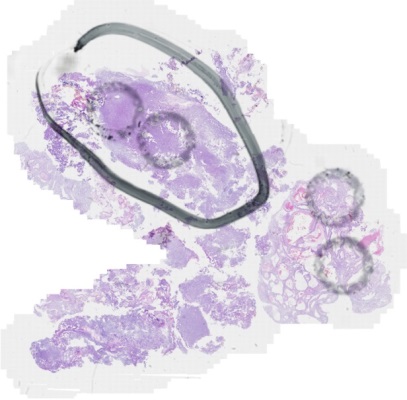


**Placement of sampling of tissue for TMA construction and for RNA extraction.** Scanned H&E stained TUR-B whole section slides for two representative tumours. TMA core placement (small rings, 1 mm in diameter) and the areas marked for macrodissection followed by RNA extraction (line drawn with marker) are indicated.

**RNA extraction**

Following macrodissection, samples were immediately deparaffinized by adding 800 µl xylene followed by a gentle vortex and gently rocking the tubes until the paraffin had visibly dissolved. Next, 400 µl ethanol was added followed by a gentle vortex, spin-down, and removal of supernatant. One more wash with 1 ml pure ethanol was performed similarly before the pellet was dried on a 55 °C heat block with open lids. The dried pellet was used as input for the HighPure FFPET RNA kit (Roche #06650775001). Only half of the lysate was used for RNA extraction, and the Proteinase K incubation was run overnight at 55 °C, but otherwise the manufacturer’s instructions were followed.

**Gene expression analysis**

RNA yield and purity was assessed by spectrometry (NanoDrop) before labelling. Cases with yield >750 ng and 260/280 nm absorbance ratios above 1.7 were considered for gene expression analysis. Older samples tended to give a lower yield and be of slightly worse median purity (see figure below) but exclusions were few for all sample years. Initially 59 RNA extracts were analyzed by capillary gel electrophoresis (Bioanalyzer). All cases had very low RIN values (< 5, and for many cases no RIN value was obtained), and this method of quality control was discontinued after 59 cases, due to the inability to identify potentially poor quality cases based on RNA size distribution. Many cases with very low average RNA fragment size that did not yield a RIN value were eventually hybridized with good results. In all, 307 samples were labelled using the SensationPlus labelling kit (Affymetrix) in batches of 24 and hybridized to single Human Gene ST 1.0 chips (Affymetrix). Of the cases selected for hybridization, none had to be excluded due to low signal in Affymetrix qc metrics, indicating high quality of included samples.


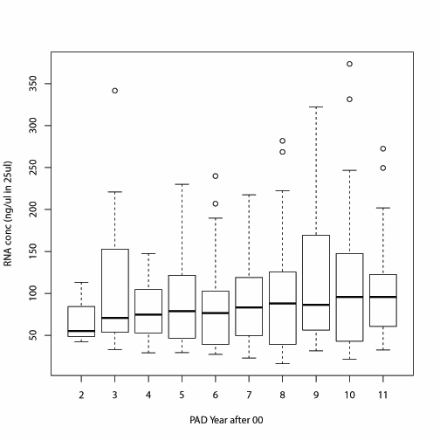

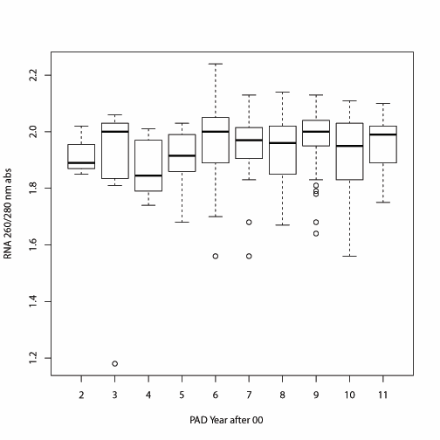


**RNA yield and quality shows slight association to sample age.** Boxplots showing RNA yield stratified by sample year (x-axis indicates year after 2000) and 260/280 nm absorbance (Nanodrop) stratified by sample year (x-axis indicates year after 2000).

**Gene expression data processing**

Raw data were normalised, low-intensity probes were removed, probes were merged by gene symbol, data were converted to log-2 scale, batch effects adjustments were performed and the data were median centered. The resulting data set contained data for 14062 genes on 307 samples. Raw data are deposited in Gene expression omnibus under GSE83586.

Pipeline:

- Quantile Normalise by batch
- Merge Batches
- Unlog
- Quantile Normalise merged data set
- Intensity filter: Remove probes with signal intensity less than the median of negative control probes for > 80% of the values.
- Unlog
- Gene symbols and EntrezID were appended using Bioconductor (hugene10stprobeset.db Version 8.3.1).
- Remove probes without genesymbol (non-coding, Ctrl. probes etc.)
- Merge probes by gene symbol (Median value)
- Log
- Apply Combat algorithm, small batch effect removed

This process was performed for 335 samples of which 307 were used in this cohort. The processed data set containing 307 cases was median centred by gene and used for all analyses. For consensus clustering, the data set was reduced by a 50% variance filter resulting in 7031 genes. Consensus clustering was also tested with a 75% Var-filter which did not substantially change the structure as assessed by the output of the ConsensusClusterPlus R package.

**Immunohistochemistry**

IHC was performed for 29 antibodies staining tumour cells and 3 antibodies for other cell types. Information on the antibodies used, primary antibody concentration, and correlation to gene expression values can be found in IHC marker table (below). No probe for NCAM1 was included in the final mRNA data set. As shown, all other antibodies used showed highly significant linear correlations between gene expression values and IHC scores, except for RXRA. The lack of correlation for RXRA is due to a weak positive overall correlation across the data, which is counteracted by a strong inverse correlation caused by the Sc/NE group. In all other groups together, the correlation observed is 0.14, but this significant correlation (p=0.0092) is completely neutered by including the Sc/NE group, in which RXRA IHC score is uniformly high, whereas *RXRA* gene expression values represent the low extreme of the data set. Furthermore, within the Sc/NE group alone, the correlation between gene expression and IHC score showed -0.27 (p=0.10) indicating a possible inverse correlation between gene and protein expression in this group. The subcellular localization (nuclear) and the staining pattern of non-tumour cells (lymphocytes stain strong nuclear positivity) are both consistent with the literature, but we cannot exclude the possibility that the antibody is non-specific (i.e. cross-reacts with some antigen specifically expressed in the Sc/NE group).

The panel of IHC markers used in this study was selected to include markers for gene expression subtypes (ERBB2, ERBB3, EGFR, FGFR3, GATA3, FOXA1, CDH1, CDH3, TP63, p-STAT3, RXRA), for proliferation (CCNB1, FOXM1), for important subtype-associated genomic events (CCND1, CDKN2A, E2F3, RB1), for urothelial differentiation states (EGFR, KRT14, KRT5, CDH3, KRT20, TP63, UPK3), and for elucidation of novel subtypes/phenotypes (EPCAM, VIM, ZEB2, TUBB2, CHGA, SYP, NCAM1), or for facilitation of detection of non-tumour cell types (CD3, CD68, ACTA2). Antibodies were tested and included if staining was positively correlated to gex-data and staining was consistent with literature and sub-cellular protein localization.

^a^ Antibody against Tyr705-P-STAT3 correlated to global STAT3 gene expression.

^b^ Change in linear correlation IHC-GEX (Fresh-Frozen tissue, Illumina HT-12) for markers analysed in Sjödahl et al. 2013 to the current study IHC-GEX (FFPE, Affymetrix Gene ST1.0)

IHC evaluation was done either on an intensity scale (0-3), or on a percentage scale (percentage positive tumour cells, 0-9, in bins of 10%), or both (See IHC Marker Table). For CCNB1, RB1 and TP63, the intensity was disregarded and only the percentage of positive tumour cells was determined (range 0-9). For all remaining markers, the intensity alone (range 0-3), or the intensity multiplied by the percentage of positive cells (range 0-3 x 0-0.9 = 0-2.7) was determined. The global structure of the data is shown in Figure S6, where the markers’ mean IHC score is shown, stratified by the deconstructed gene expression phenotypes (Uro, green; UroB, brown; GU, Blue; SCCL, red; Mes-Inf, orange; Sc/NE, purple). Fifteen markers were analysed also in a previous study (Sjödahl et al., 2013; citation [8] in the main text) where sampling was done differently. In that study, a fresh-frozen tissue sample was taken from the exophytic part of the tumour (TUR-B) for gene expression analysis using Illumina HT-12 arrays, whereas the TMA was constructed from the deep part used for pathological analysis. This study included both non muscle-invasive (NMI) and muscle-invasive (MI) urothelial cancers. Thus, there are two differences between these two studies; i) The current study included only advanced cases, whereas the 2013 study included both NMI and MI cases. ii) The current study sampled tissue as close as possible to the TMA cores, whereas the 2013 study sampled the gene expression and TMA tissue from different parts of the tumour. It is most likely this improved sampling has led to the sometimes dramatically increased correlation between gene expression values and IHC score. Notably, the only gene for which correlation did not improve was Cyclin B1. Proliferation varies across a much narrower span in the current MI-only cohort than in a mixed NMI-MI cohort, and this is likely causing the lack of improvement of correlation for this marker.

**Assignment of tumour-cell phenotypes based on IHC data**

Cellular phenotypes of advanced bladder cancer was defined by calculating *definition scores* as follows:

**Uro/UroB:** CCND1+, FGFR3+, RB1+, p16-

**Genom. Unstable:** CCND1-, FGFR3-, RB1-, p16+

**Mes-like:** VIM+, ZEB2+, EPCAM-, E-Cad-

**Basal/SCC-like:** KRT5+, KRT14+, GATA3-, FOXA1-

**Sc/NE-like:** TUBB2B+, EPCAM+, E-Cad-, GATA3-

To classify a sample, each of the 13 individual markers’ score was determined as described above and the scores were divided by the range-maximum to normalise scores recorded as percentages vs. those recorded as intensities. Thus the score for RB1 (0-9) was divided by 9, and the score for all other markers (0-3) were divided by 3. The definition scores were then calculated by adding/subtracting each normalised marker score. A case was considered positive for a molecular phenotype if the definition score was higher than 0.6. Next, the unambiguous assignment of tumour-cell phenotype was determined as the highest positive (>0.6) value of the **Mes-like**, **Basal/SCC-like**, and **Sc/NE-like** definition scores. If neither of these scores was above 0.6, the case was classified as **Uro/UroB** if the definition score was positive (>0.6). If the case is below 0.6 it is defined as **Genomically Unstable** (this phenotype is thus defined as opposite to that of the **Uro/UroB**). The details of classification are not to be regarded as a finished classification algorithm set in stone. If IHC evaluation is performed on whole-sections or otherwise differently in another lab, the calculations should be adapted accordingly, and it should be recognised that increased accuracy (at the expense of applicability) may be reached by adding more subtype specific markers.

**SUPPLEMENTAL REFERENCES**

27. Blaveri E, Simko JP, Korkola JE*, et al.* Bladder cancer outcome and subtype classification by gene expression. *Clin Cancer Res* 2005; **11**: 4044-4055.

28. GTEx Consortium. Human genomics. The Genotype-Tissue Expression (GTEx) pilot analysis: multitissue gene regulation in humans. *Science* 2015; **348**: 648-660.

29. Moch H, Humphrey PA, Ulbright TM, *et al*. Classification of Tumors of the Urinary System and Male Genital Organs. Lyon, France: International Agency for Research on Cancer; 2016.
