# Supplementary figures and images for: Molecular classification of urothelial carcinoma: global mRNA classification versus tumour‐cell phenotype classification
Source: J Pathol. 2017 Mar 28;242(1):113–25. doi: 10.1002/path.4886 (PMC5413843; doi:10.1002/path.4886)

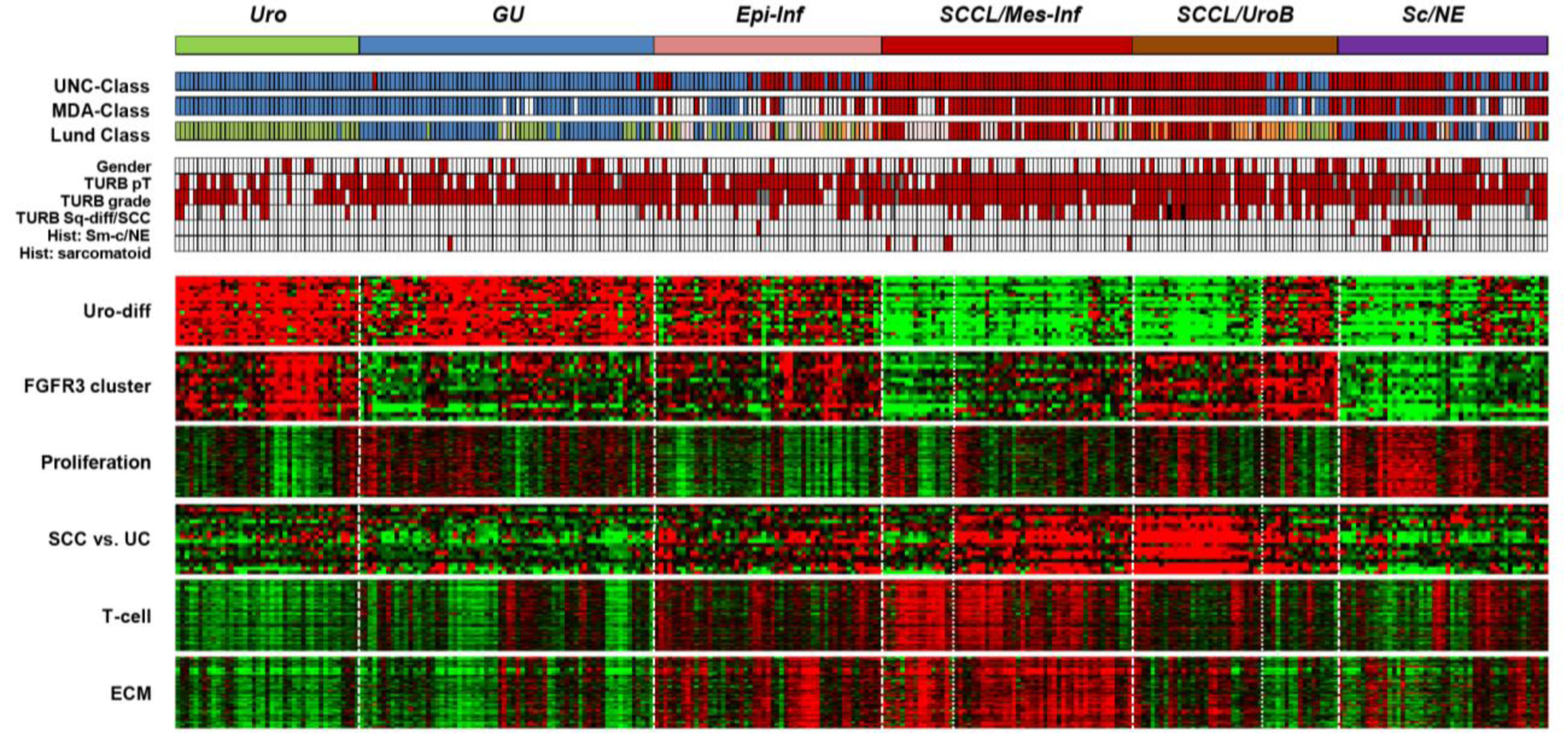

Supplement: Supplementary file 3 — Supplementary Figure S1 Consensus clustering of global mRNA expression from cystectomized patients (TUR‐B samples). Obtained tumour clusters are indicated on top. Subtype labels are adapted from Aine et al., 2015 15. Subtype classification are according to UNC‐class; blue, luminal; dark red, basal (Damrauer et al., 2014 10). MDA‐class; blue, luminal; white, TP53‐like; dark red, basal (Choi et al., 2014 9). Lund class; green, Uro; blue, GU; light brown, UroB; dark red, SCC‐like; white, infiltrated (Sjödahl et al., 2012 7). Clinical variables. Gender, white, male; dark red, female. TUR‐B pathological stage, dark red, ≥T2; white < T2. TUR‐B pathological grade, dark red, G3; white, <G3; grey, Gx. Squamous differentiation, dark red, sings of squamous differentiation; black, pathological SCC tumours. Small cell or neuroendocrine histology, dark red, presence of Sc/NE histology. Sarcomatoid histology, dark red presence of sarcomatoid histology. Heatmaps of gene expression are depicted in red (high), and green (low), colour scale. Heatmaps for genes signatures correspond to the urothelial differentiation signature (Uro‐diff), genes associated with FGFR3 mutation/overexpression (FGFR3 cluster), late cell cycle (proliferation) signature, genes upregulated in squamous cell carcinoma of the urothelium (SCC vs. UC, Blaveri et al., 2005 [27]), genes expressed by T‐cells (T‐cell), and gene related to extra cellular matrix (ECM). Citation numbers refer to the main text. [file PATH-242-113-s009.tif]

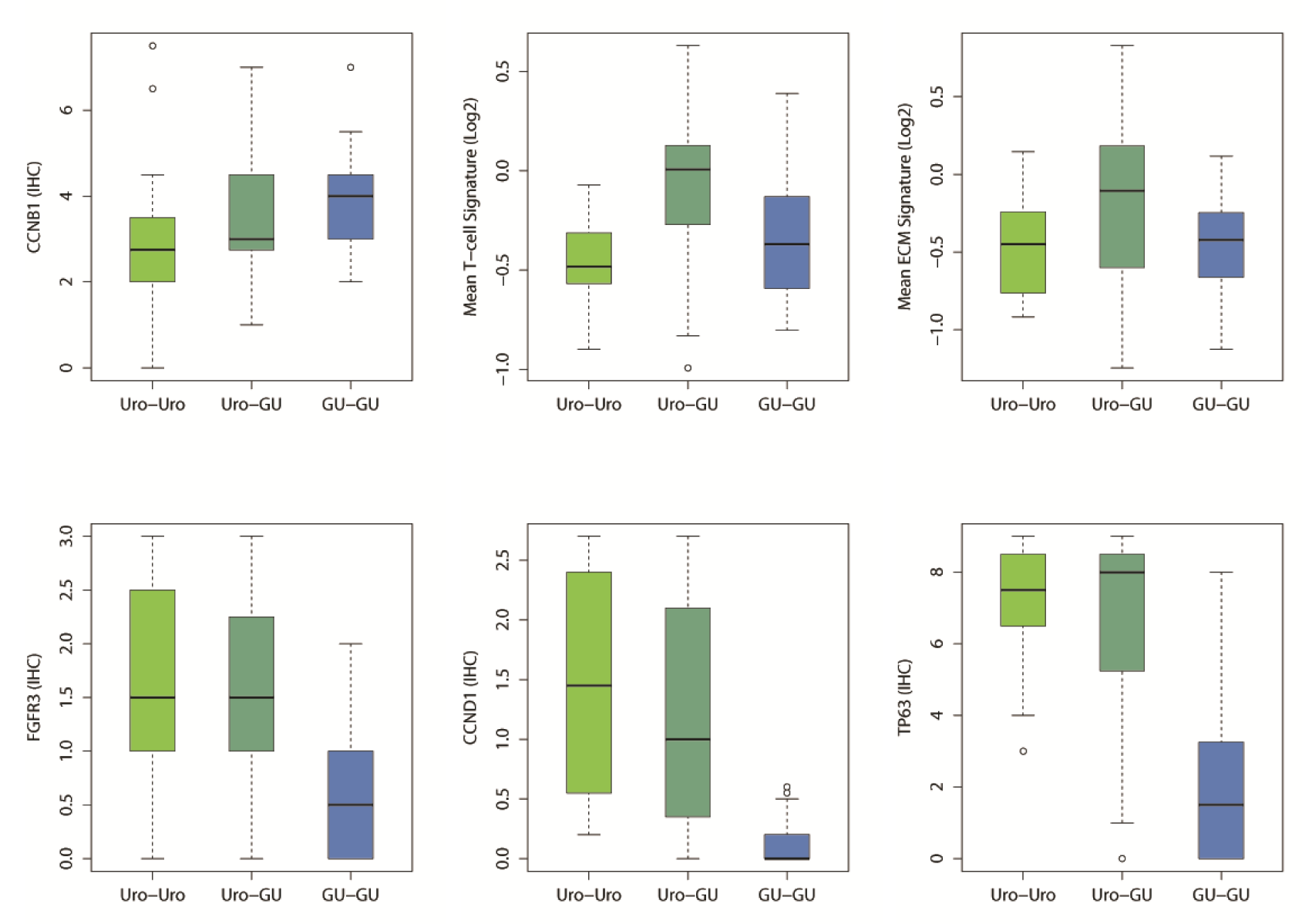

Supplement: Supplementary file 4 — Supplementary Figure S2 Cases with Uro phenotype that cluster in the GU consensus cluster retain their Uro molecular profile but proliferation, T‐cell and ECM signals are affected. For each plot, the y‐axis label indicates the data type (IHC, or mRNA‐signature). Cases with Uro phenotype in the GU consensus cluster (dark green) are shown to have increased CCNB1 staining (proliferation), T‐cell‐, and ECM‐Signatures, compared to their Uro ‐ Uro counterpart (light green). For canonical Uro markers (FGFR3, CCND1, TP63), however, there was no difference, confirming their molecular profile as Uro tumours. Tumours with a GU phenotype lack expression of these canonical markers. [file PATH-242-113-s004.tif]

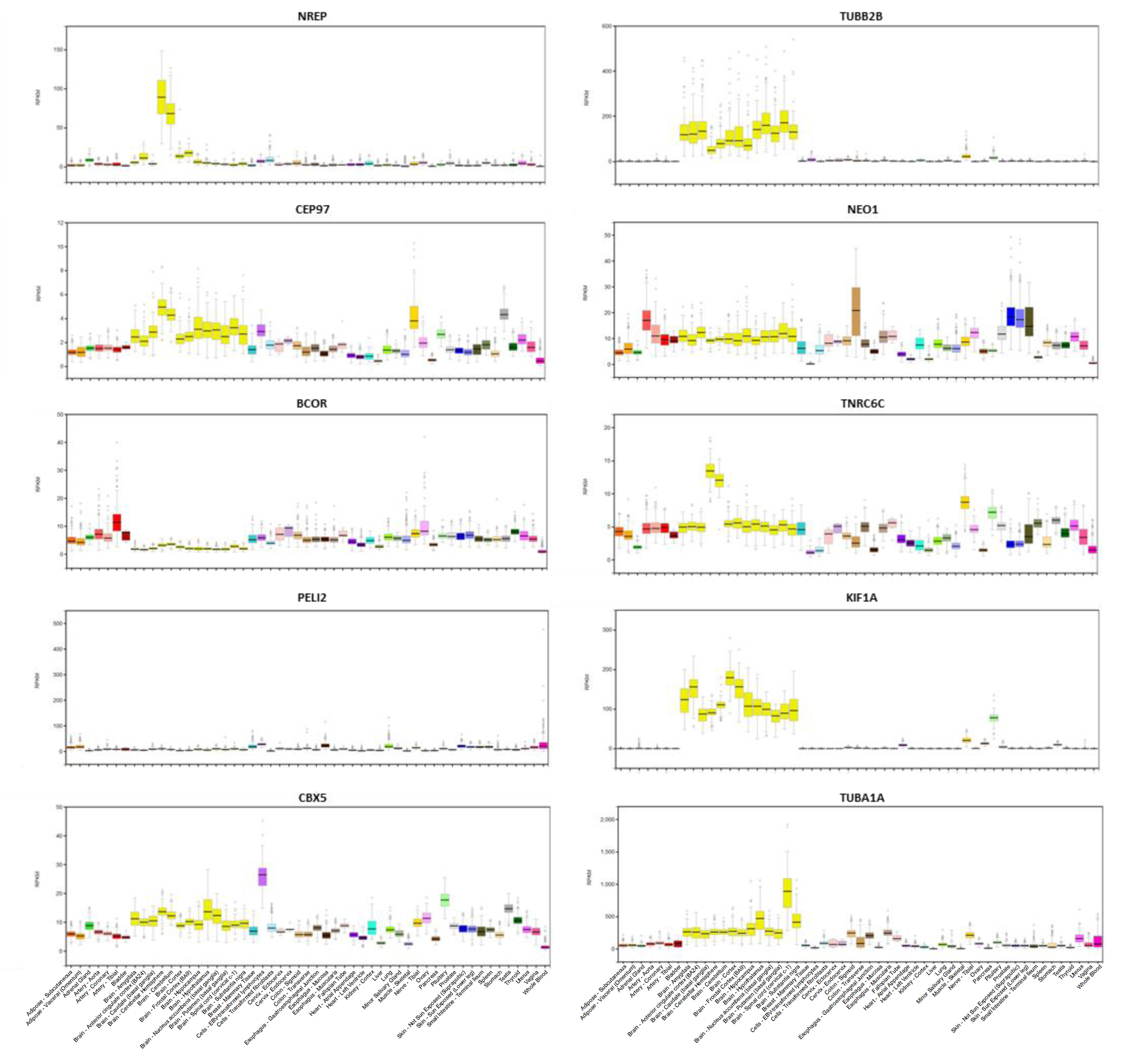

Supplement: Supplementary file 5 — Supplementary Figure S3 Top 10 upregulated genes in Sc/NE – Sc/NE group (compared to Sc/NE – GU) frequently have a normal tissue expression pattern restricted to neuronal/endocrine tissues. SAM analysis was perfomed comparing the two groups and the top 10 upregulated genes were queried in the gTEX database (RNA‐Seq data from normal tissues, gtexportal.org, gTEX consortium, 2015 [28]). Boxplots of FPKM values; Yellow boxes represent brain tissues of different anatomical locations, light green represents pituitary gland, orange represents tibial nerve. Several of the most upregulated genes show normal expression restricted to neuronal/endocrine tissues. Citation numbers refer to the main text. [file PATH-242-113-s012.tif]

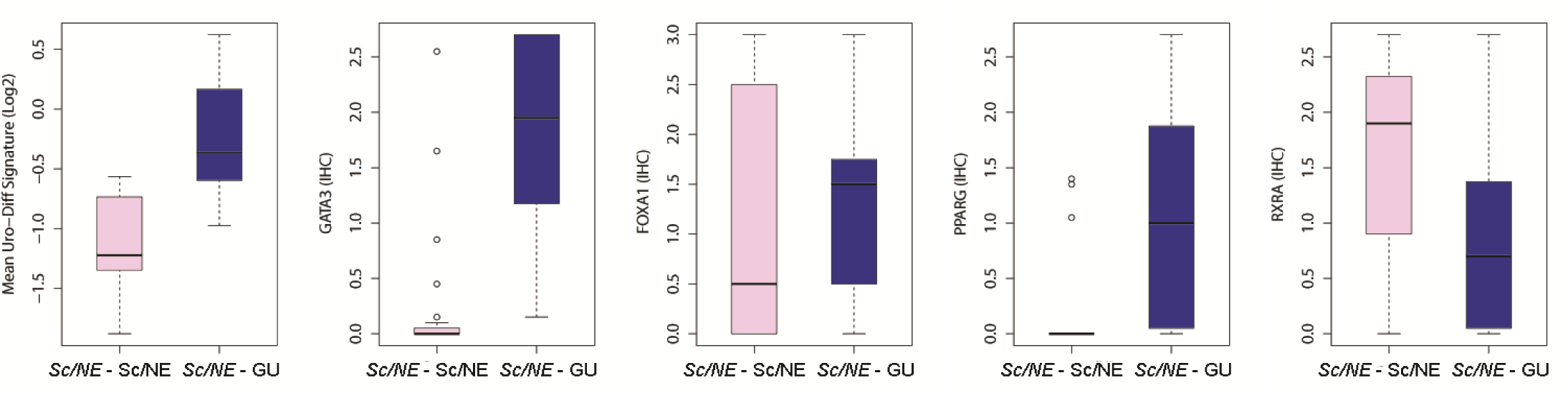

Supplement: Supplementary file 6 — Supplementary Figure S4 Cases with GU phenotype that cluster in the Sc/NE consensus cluster retain expression of the UroDiff‐signature (Mean mRNA) and key factors (IHC). For each plot, the y‐axis label indicates the data type (IHC, or mRNA‐signature). Cases with GU phenotype in the Sc/NE consensus cluster (dark blue) are shown to have retained expression of Uro‐diff signature (left) and tumour cell expression of GATA3, FOXA1, and PPARG. Curiously RXRA (IHC) indicated high levels in the cases with Sc/NE phenotype, which was not the case for the RXRA values in the gene expression data set. Thus, Sc/NE ‐ Sc/NE cases lose urothelial differentiation signature, except for possibly RXRA protein expression, whereas the cases with GU phenotype in the same consensus cluster do not. [file PATH-242-113-s001.tif]

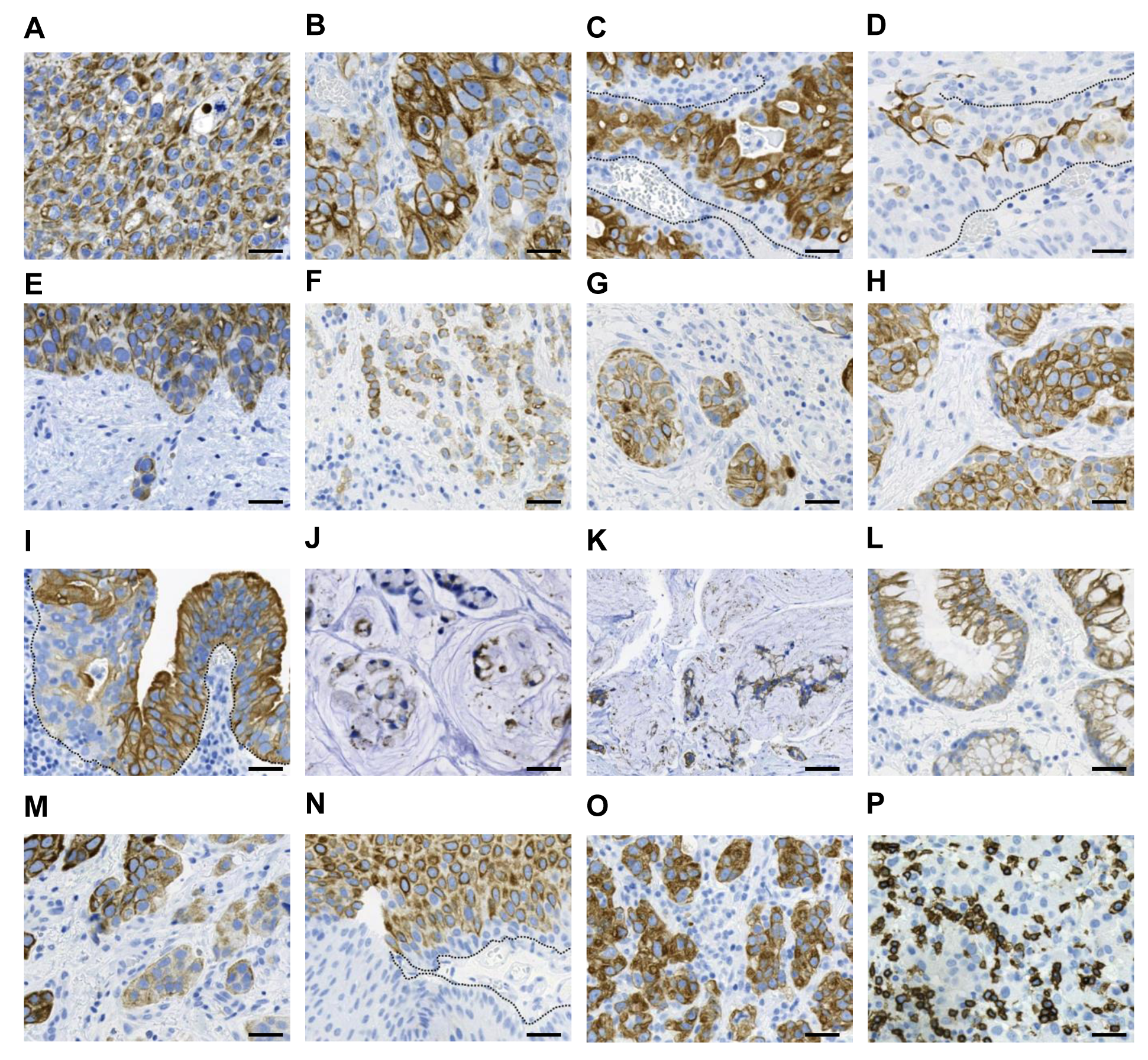

Supplement: Supplementary file 7 — Supplementary Figure S5 Protein expression patterns of KRT20 in advanced urothelial carcinoma is de‐regulated and inconsistent with normal urothelial differentiation. (A) KRT20 positive strong cytoplasmic staining. (B) KRT20 positive strong membraneous staining. (C) KRT20 positive case where basal cells are negative. All cells distal to the tumour‐stroma interface are positive. (D) KRT20 positive case where few cells most distal to the tumour‐stroma interface are positive. (E) KRT20 positive case where bulk tumour, basal cells, and a lamina propria invading nest are positive. (F) KRT20 positive case with an infiltrative invasive growth pattern. (G) KRT20 positive case with a nodular invasive growth pattern. (H) KRT20 positive case with a trabecular invasive growth pattern. (I) Dysplastic urothelium with highly variable KRT20 staining. (J‐K) KRT20 positive mucinous tumours with signet‐ring morphology. Scale bars indicate 100 µm. (L) Sample including intestinal metaplasia, showing KRT20 positivity. (M) KRT20 positive case with heterogeneous expression (non‐clonal appearance). (N) KRT20 positive case with heterogeneous expression (clonal appearance). (O) KRT20 positive strongly lymphocyte‐infiltrated Genomically Unstable tumour (Sc/NE ‐ GU). (P) CD3 staining of the same case as in (O), showing dense lymphocyte infiltration. Scale bars for (A‐I) and (L‐O) indicate 20 µm. [file PATH-242-113-s008.tif]

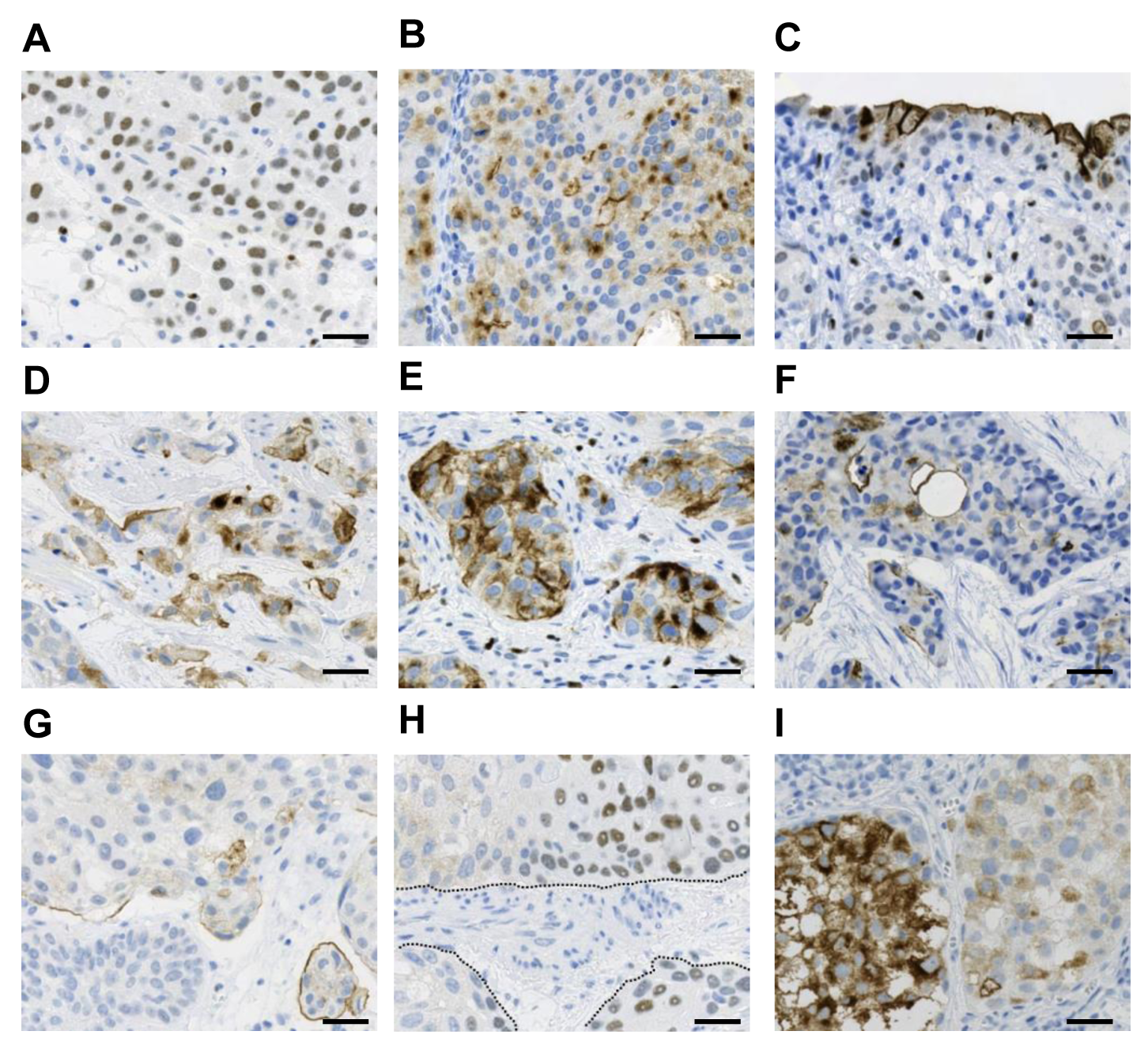

Supplement: Supplementary file 8 — Supplementary Figure S6 Protein expression of UPK3 in advanced urothelial carcinoma is de‐regulated and inconsistent with normal urothelial differentiation. (A) UPK3 positive case showing strong nuclear staining. (B) UPK3 positive case showing cytoplasmic staining. (C) Strong apical UPK3 positivity in luminal cells that lack normal urothelial organization. (D) UPK3 positive case with an infiltrative invasive growth pattern. (E) UPK3 positive case with a nodular invasive growth pattern. (F) UPK3 positive case with a trabecular invasive growth pattern. (G) Tumour nests displaying a ‘pushing‐border’ growth pattern with UPK3 positivity along the nests' circumference, resulting in an inverted staining pattern (cells closest to tumour‐stroma interface positive). (H) UPK3 positive case with heterogeneous expression (non‐clonal appearance). (I) UPK3 positive case with heterogeneous expression (clonal appearance). Scale bars indicate 20 µm. [file PATH-242-113-s011.tif]

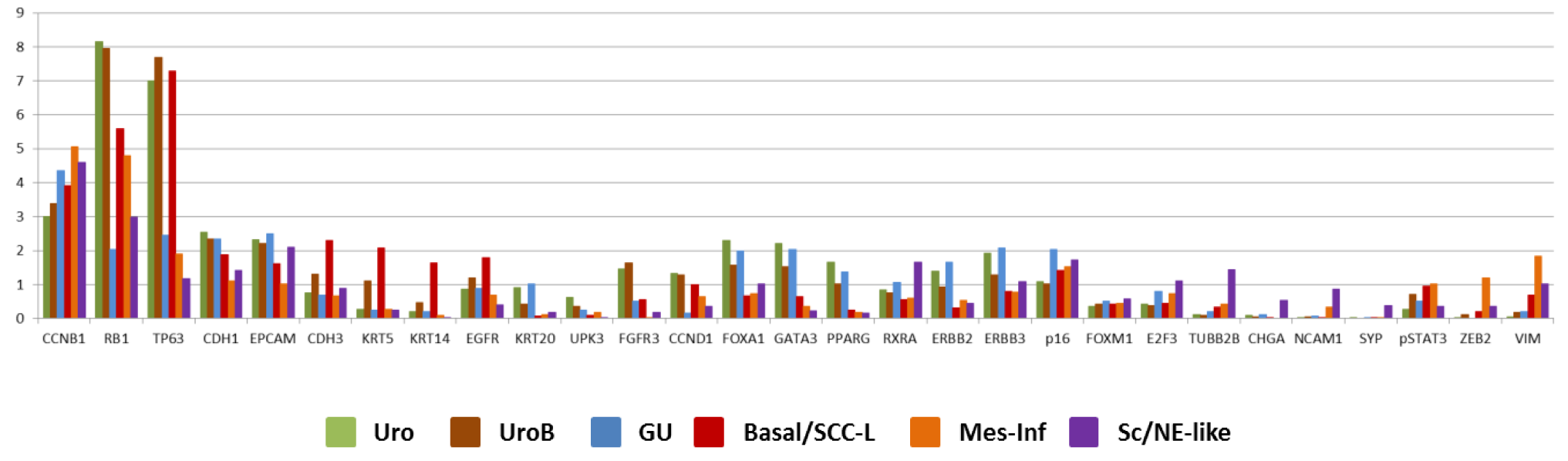

Supplement: Supplementary file 9 — Supplementary Figure S7, See also Supplementary Figure 8. IHC data from 29 markers showing differential expression in tumour cells were considered in establishing tumour‐cell phenotype definitions. Barplots showing the mean IHC scores for the deconstructed gene expression phenotypes including UroB, as we initially intended to produce a separate IHC definition also for UroB. Uro includes Uro cases from the Uro, GU, and Epi‐Inf clusters. GU includes GU cases from the GU, Epi‐Inf, and Sc/NE clusters. Basal/SCCL includes Basal/SCC‐like cases from the SCCL/Mes‐Inf and SCCL/UroB clusters. [file PATH-242-113-s005.tif]

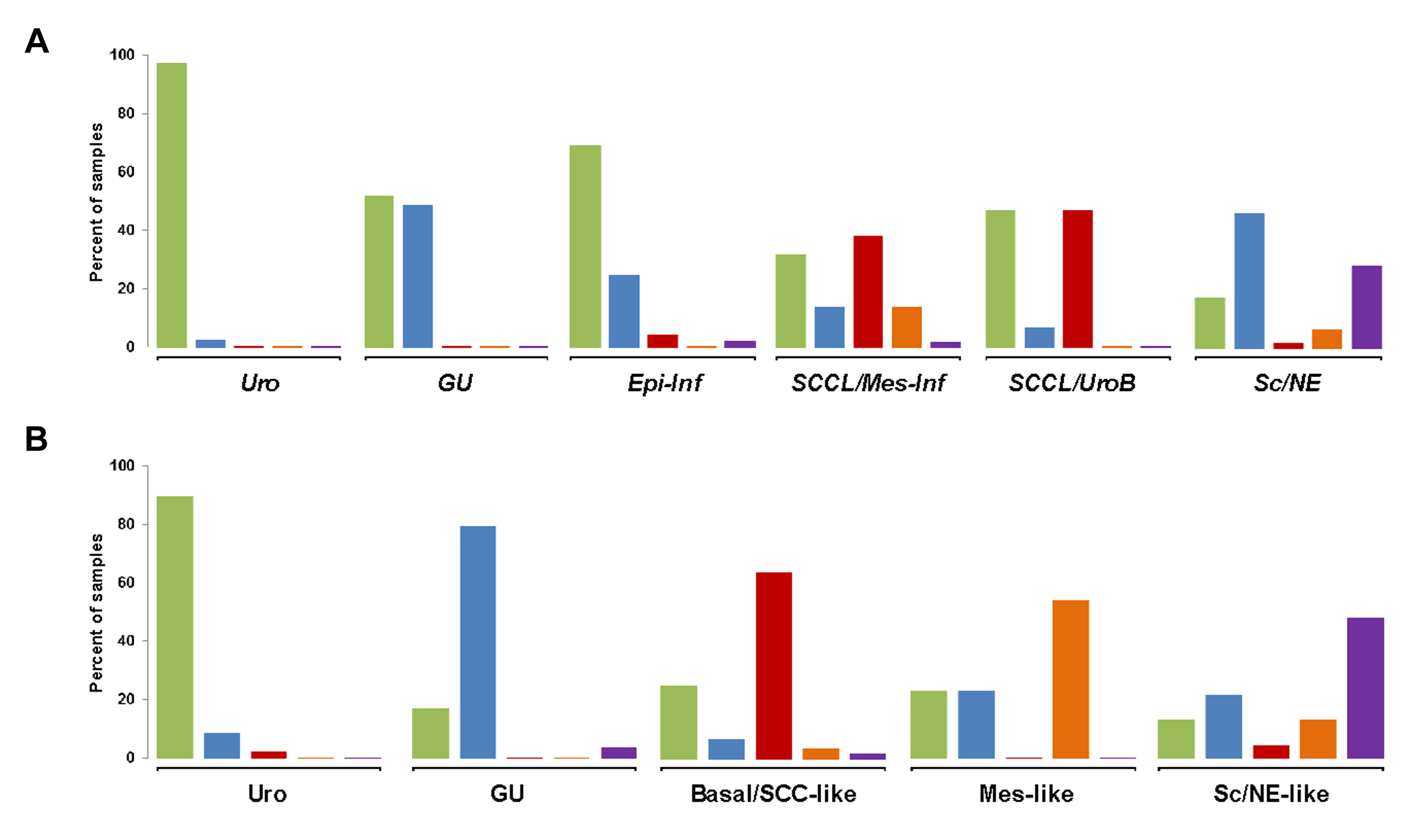

Supplement: Supplementary file 10 — Supplementary Figure S8, Concordance of tumour‐cell phenotype with consensus clusters and deconstructed subtypes. (A) IHC classification based on definitions in Figure 6 of tumours, grouped by the consensus clusters Urothelial‐like (Uro), Genomically Unstable (GU), Epithelial Infiltrated (Epi‐Inf), SCC‐like/Mesenchymal Infiltrated (SCCL/Mes‐Inf), SCC‐like/Urothelial‐like B (SCCL/UroB), and the Small‐cell/Neuroendocrine (Sc/NE). (B) IHC classification based on definitions in Figure 6 of tumours, grouped according to deconstructed tumour subtypes as determined by examination of mRNA profiles. Uro, composed of Uro cases from the Uro, GU, and Epi‐Inf consensus clusters, and UroB cases from the SCC‐like/UroB consensus cluster. GU, composed of GU cases from the GU, Epi‐Inf and Sc/NE consensus clusters. SCC‐like, composed of Basal/SCC‐like cases from the SCC‐like/Mes‐Inf and SCC‐like/UroB consensus clusters. Mes‐Inf and Sc/NE subclusters as defined in the text. [file PATH-242-113-s006.tif]
