# Supplementary material for: Molecular classification of urothelial carcinoma: global mRNA classification versus tumour‐cell phenotype classification
Source: J Pathol. 2017 Mar 28;242(1):113–25. doi: 10.1002/path.4886 (PMC5413843; doi:10.1002/path.4886)
Supplement: Supplementary file 11 — Table S1. Histological variants included in the consecutive series of 307 tumours according to the 2016 WHO classification of invasive bladder tumours. Table S2. The pathological stage and grade based on uro‐pathologist re‐evaluation of TUR‐B specimens for the consecutive series of 307 tumours. Table S3. Differentially expressed genes between Mes‐Inf and Basal/SCC‐like, between UroB and Basal/SCC‐like, and between Sc/NE and GU. Table S4. Comparison of tumour‐cell phenotypes (applied IHC definitions) to global mRNA consensus clusters (top). Table S5. Comparison of tumour‐cell phenotypes (applied IHC definitions) to deconstructed mRNA clusters (as described in text). [file PATH-242-113-s003.docx]

| **Histology** | **Numbers** |
| --- | --- |
| Pure urothelial carcinoma | 240 |
| Infiltrating urothelial with divergent differentiation (squamous differerentiation, glandular differentiation, and trophoblastic differentiation) | 42 |
| Micropapillary | 2 |
| Nested, including large nested | 2 |
| Microcystic | 1 |
| Sarcomatoid | 7 |
| Poorly differentiated and small cell carcinoma | 7 |
| Giant cell carcinoma | 1 |
| Pure squamous cell carcinoma | 2 |
| Pure adenocarcinoma | 3 |

**Table S1. Histological variants included in the consecutive series of 307 tumours according to the 2016 WHO classification of invasive bladder tumors [29].**

| **Pathological stage** | **Numbers** | **Pathological grade** | **Numbers** |
| --- | --- | --- | --- |
| **pTa** | **13** | **G1** | **0** |
| **pT1** | **44** | **G2** | **28** |
| **pT2** | **243** | **G3** | **266** |
| **pTis** | **1** | **Gx** | **13** |
| **pTx** | **5** |  |  |

**Table S2. The pathological stage and grade based on uro-pathologist re-evaluation of TUR-B specimens for the consecutive series of 307 tumours.**

**Table S3. Differentially expressed genes between Mes-Inf and Basal/SCC-like, between UroB and Basal/SCC-like, and between Sc/NE and GU.**

Top 100 genes are shown for each comparison. Comparisons are made only between sub-clusters within consensus clusters. False discovery rate controlled at q<0.01. FC, fold change.

|  | ***Uro*** | ***GU*** | ***Epi-Inf*** | ***SCCL/ Mes-Inf*** | ***SCCL/ UroB*** | **Sc/NE** |
| --- | --- | --- | --- | --- | --- | --- |
| **Uro** | 38 | 32 | 33 | 16 | 21 | 8 |
| **GU** | 1 | 30 | 12 | 7 | 3 | 21 |
| **SCC-like** |  |  | 2 | 19 | 21 | 1 |
| **Mes-like** |  |  |  | 7 |  | 3 |
| **Sc/NE-like** |  |  | 1 | 1 |  | 13 |

**Table S4. Comparison of tumour-cell phenotypes (applied IHC-definitions) to global mRNA consensus clusters (top).**

|  | **Uro** | **GU** | **Basal/**  **SCCL** | **Mes-like** | **Sc/NE-like** | **Spec.** |
| --- | --- | --- | --- | --- | --- | --- |
| **Uro** | **85** | 9 | 15 | 3 | 3 | 0.74 |
| **GU** | 8 | **42** | 4 | 3 | 5 | 0.68 |
| **SCC-like** | 2 |  | **38** |  | 1 | 0.93 |
| **Mes-like** |  |  | 2 | **7** | 3 | 0.58 |
| **Sc/NE-like** |  | 2 | 1 |  | **11** | 0.79 |
| **Sens.** | 0.89 | 0.79 | 0.63 | 0.54 | 0.48 |  |

**Table S5. Comparison of tumour-cell phenotypes (applied IHC-definitions) to deconstructed mRNA clusters (as described in text).**

Top row: Uro includes Uro cases from the *Uro*, *GU*, *Epi-Inf* and *SCCL/UroB* clusters. GU includes GU cases from the *GU, Epi-Inf* and *Sc/NE* clusters. Basal/SCCL includes Basal/SCC-like cases from the *SCCL/Mes-Inf* and *SCCL/UroB* clusters. Bold numbers indicate agreement between tumour-cell phenotype and the corresponding mRNA phenotypes. Sensitivity and specificity are calculated based on correspondence of the tumour-cell phenotypes to the gene expression phenotypes as described.
